# Identification of mitochondrial permeability transition-related lncRNAs as quantitative biomarkers for the prognosis and therapy of breast cancer

**DOI:** 10.3389/fgene.2025.1510154

**Published:** 2025-03-26

**Authors:** Zhongshu Lin, Xinlu Wang, Guanxiang Hua, Fangmin Zhong, Wangxinjun Cheng, Yuxiang Qiu, Zhe Chi, Huan Zeng, Xiaozhong Wang

**Affiliations:** ^1^ Jiangxi Province Key Laboratory of Immunology and Inflammation, Jiangxi Provincial Clinical Research Center for Laboratory Medicine, Department of Clinical Laboratory, The Second Affiliated Hospital, Jiangxi Medical College, Nanchang University, Nanchang, Jiangxi, China; ^2^ School of Biological and Behavioural Science, Queen Mary University of London, London, United Kingdom; ^3^ Queen Mary College, Nanchang University, Nanchang, China; ^4^ School of Public Health, Jiangxi Medical College, Nanchang University, Nanchang, Jiangxi, China

**Keywords:** transcriptomics, RNA, breast cancer, long non-coding RNA, tumor, mitochondrial permeability transition

## Abstract

Breast cancer (BC) continues to pose a global health threat and presents challenges for treatment due to its high heterogeneity. Recent advancements in the understanding of mitochondrial permeability transition (MPT) and the regulatory roles of long non-coding RNAs (lncRNAs) offer potential insights for the stratification and personalized treatment of BC. Although the association between MPT and lncRNAs has not been widely studied, a few research studies have indicated a regulatory impact of lncRNAs on MPT, further deepening the understanding of the tumor. To identify reliable biomarkers associated with MPT for managing BC, bulk RNA-seq data of MPT-related lncRNAs acquired from The Cancer Genome Atlas (TCGA) and the Genotype-Tissue Expression (GTEx) project were utilized to assess BC patients. A scoring system, termed the MPT-related score (MPTRscore), was developed using LASSO-Cox regression on data from 1,029 BC patients from TCGA-BRCA. Meanwhile, the superior prognostic accuracy of the MPTRscore was demonstrated by comparing it with biomarkers, including PAM50 subtyping for standardization. Subsequently, a clinical prediction model was created by incorporating the MPTRscore and clinical variables. This analysis revealed two distinct MPTRscore groups characterized by different biomolecular processes, tumor microenvironment (TME) patterns, and clinical outcomes. The MPTRscore was further investigated through unsupervised consensus clustering of TCGA-BRCA based on MPTRscore-related prognostic genes. Additionally, the MPTRscore was identified as an independent prognostic factor for BC and showed guiding utility in immunotherapy and chemotherapy response. Specifically, patients with a low MPTRscore exhibited better prognosis and treatment responses compared to those with a high MPTRscore. Significantly, the relevance of clustering results and MPTRscore was found to be mediated by lncRNA transcript RP11-573D15.8-018. In conclusion, MPTRscore-related clusters were identified in BC, and an integrative score was developed as a biomarker for predicting BC prognosis and therapeutic response. Additionally, molecular interactions underlying the relationship between MPTRscore-related clusters and MPTRscore were uncovered, proving insights for BC stratification. These findings may aid in prognosis determination and therapeutic decision-making for BC patients.

## 1 Introduction

Breast cancer (BC) was the most commonly diagnosed cancer worldwide in 2020 and remains a major public health concern due to its high prevalence and mortality rate. BC affects millions globally, with over 2.3 million new cases recorded annually, leading to significant morbidity and mortality (Sung et al., 2021). Despite advancements in treatment, accurately predicting the prognosis of BC remains difficult due to its heterogeneous nature: BC can be categorized into five principal molecular subtypes using PAM50 technology (Xia et al., 2023).

The mitochondrial permeability transition (MPT) involves the formation of a highly conductive pore within the inner mitochondrial membrane, which is induced by excessive Ca^2+^ and reactive oxygen species (ROS). This transition leads to notable changes in both the structure and function of mitochondria, affecting various enzyme activities that control mitochondrial respiration and ATP synthesis (Bauer and Murphy, 2020). MPT is characterized by the blunt increase in permeability of the mitochondrial membranes, and it can be critical to miscellaneous cellular physiological processes, such as the control of mitophagy and the invigoration of apoptosis or necrosis. The MPT can be controlled by the mitochondrial permeability transition pore (mPTP), a complex located at the inner and outer mitochondrial membranes (Bonora et al., 2022). Interestingly, MPT is related to cancer prognosis and has been considered an available target for cancer treatment (Dalla Via et al., 2014).

Long non-coding RNAs (lncRNAs) are RNA molecules longer than 200 nucleotides that do not encode proteins (Bhan et al., 2017). lncRNAs play various significant roles in regulating transcriptional and post-transcriptional modifications. They are closely associated with regulated cell death, which is strongly related to cancer prognosis: lncRNAs have been shown to affect apoptosis, autophagy, and necrosis (Rossi and Antonangeli, 2014; Liu et al., 2018; Wang et al., 2016). Certain lncRNAs have been identified to function by altering gene expression through binding to the 3′-untranslated regions (3′-UTRs) of mRNAs, acting as enhancers, or interacting with transcription factors and miRNAs to modulate gene networks related to cancer (Yang et al., 2022). In BC, lncRNAs have gained attention for their potential as biomarkers for diagnosis and prognosis (Zhu et al., 2021). The lncRNA HOX transcript antisense intergenic RNA (HOTAIR) has been found to interact with the polycomb repressive complex 2 (PRC2), influencing epigenetic modifications and affecting gene expression patterns involved in BC progression (Mozdarani et al., 2020).

LncRNAs’ association with MPT has not been widely studied. However, several studies have revealed a subtle regulatory role of lncRNAs on MPT. For instance, a recent research study found that the lncRNA OIP5-AS1 demonstrated an anti-mitochondrial-apoptosis function in HL-1 cells by suppressing the opening of the mPTP (Niu et al., 2024). Additionally, a few studies have successfully identified MPT-related lncRNAs (MPTRLs) using bioinformatics tools (Huang et al., 2023). There have been no studies linking BC to MPT-driven necrosis. This study aims to identify the elusive connection between MPT-driven necrosis-related RNA and BC by building a prognostic model and analyzing the drug sensitivity of treatments currently given to BC based on the model constructed.

To find possible biomarkers for BC stratification related to MPTRLs, in this study, a stable 7-MPTRL-based risk score was constructed via LASSO-Cox regression. Furthermore, we identified high and low MPT-related score (MPTRscore) groups with differences in molecular processes, tumor microenvironment (TME) patterns, and prognosis. Then, a clinical prediction model integrating the MPTRscore and other clinical parameters was built for the prognosis prediction of BC patients. Moreover, a potential prospect of the MPTRscore for chemotherapeutic and immunotherapeutic sensitivity prediction was demonstrated. Based on the MPTRscore, two MPTRscore-related clusters were identified. Furthermore, the lncRNA transcript RP11-573D15.8-018 was found to be the key linkage between MPTRscore-related clusters and MPTRscore by constructing an lncRNA–RNA interaction network.

## 2 Materials and methods

We integrated multi-omics data analysis, machine learning, and experimental validation to systematically identify BC-associated MPTRLs and construct a prognostic risk model. First, transcriptomic data from The Cancer Genome Atlas (TCGA)-BRCA and Genotype-Tissue Expression (GTEx) cohorts were processed. Differential expression and correlation analyses were performed on the data to pinpoint MPTRLs linked to BC pathogenesis, followed by the development of a risk score model predictive of patient survival. Furthermore, a nomogram was constructed incorporating the risk score, age, and T and N stages to predict prognosis. Its performance was assessed through statistical analysis. Pathway enrichment, tumor immune cell analyses, and pharmacogenomic profiling were performed on the low- and high-risk groups. PAM50 classification subtypes were also analyzed to unravel the relationship between MPTRscore and PAM50 classification subtypes. MPTRscore-related prognostic genes were selected using differential expression analysis and univariate Cox regression, with patients divided into two clusters using unsupervised clustering. Pharmacogenomic profiling, single-cell resolution clustering, and the construction of an lncRNA–RNA interaction network were further conducted based on the clustering groups. Ultimately, RT-qPCR and RNA knockdown experiments were performed to validate key lncRNAs.

### 2.1 Acquisition of BC-associated MPT-related lncRNAs

To extract the BC-associated MPT-related lncRNAs, it is necessary to prepare gene expression profiles of breast cancer samples and genes related to MPT. The BC transcriptome sequencing data were downloaded in logTPM from TCGA-BRCA of the TCGA database using the R package “TCGAbiolinks” along with the attached clinical information (Colaprico et al., 2015). In addition, integrated transcriptome sequencing data of BC and normal breast samples from TCGA and GTEx databases, coupled with clinical information in counts, was downloaded from UCSC Xena (https://xena.ucsc.edu/); the batch effect between the two databases had already been eliminated, followed by the extraction of data from primary BC patients (Goldman et al., 2020). The conversion relationship between the gene symbol and gene ID of lncRNAs can be acquired in supplementary data. When multiple probes were matched to the same gene symbol, the expression level of that gene was taken as the mean value. In order to alleviate statistical error, data of BC patients without overall survival (OS) values or with OS values smaller than 30 days were deleted. With the attached survival data, 1,129 patients were selected and separated into the train and test risk groups arbitrarily using the caret R package, with a 4:1 ratio (Kuhn, 2008).

### 2.2 Identification of BC-associated MPTRLs

To further identify BC-associated MPTRLs, the relationship between the expression of MPT-related genes and a set of lncRNAs in breast cancer samples was first calculated. Only those lncRNAs that both significantly correlated with MPT-related genes and were differentially expressed between tumor and adjacent normal tissues were considered. Differentially expressed lncRNAs were identified through differential gene expression (DEG) analysis of transcriptome sequencing data downloaded from TCGA and GTEx. DEG analysis is a technique used to identify genes expressed at significantly different levels between tumor and normal samples. Differential lncRNA expression analysis was conducted using specific criteria: a false discovery rate (FDR) of less than 0.001 and a log fold change (logFC) of at least 0.585. Based on these thresholds, 323 differentially expressed lncRNAs were identified. A volcano plot was generated to illustrate the differences in lncRNA expression between tumor tissues and adjacent normal tissues. The gene set associated with MPT, comprising 39 genes, was sourced from a recent study, which can also be downloaded from supplementary data (Liu et al., 2023). Correlation analysis was conducted to assess the relationship between 39 MPT-related genes and the differentially expressed lncRNAs identified. Subsequently, 1,478 lncRNAs exhibited Pearson correlation coefficients greater than 0.3 and a significance level of p < 0.01, with MPT-related genes classified as MPTRLs. A correlation coefficient >0.3 and a p-value threshold were applied to ensure statistical significance and biological relevance, thus reducing noise and improving the robustness of the selection process.

### 2.3 Establishment of the risk model

After acquiring MPTRLs, a risk model was developed by quantifying and integrating the expression levels of multiple MPTRLs. First, we performed univariate Cox regression for each MPTRL to evaluate its individual association with OS, using a significance threshold of p < 0.05. The univariate Cox regression allowed us to perform regression between the expression levels of lncRNAs and overall survival outcomes and screen for lncRNAs that were expressed to be significantly associated with survival outcomes. Next, to reduce potential multicollinearity and avoid overfitting, we applied LASSO regression with 10-fold cross-validation to the significant lncRNAs from the univariate analysis. The LASSO method automatically selected the most predictive lncRNA features by carrying out regression between the expression levels of lncRNAs and survival outcomes, shrinking the coefficients of less informative lncRNAs to 0. The lncRNAs with non-zero coefficients were incorporated into a multivariate Cox regression model, which works similarly to a univariate Cox regression model but considers covariates such as age and TNM staging to exclude potential confounders. The optimal lambda value used to train the model, namely, the regularization parameter in the regression function, was determined through cross-validation. The final risk score model was based on the coefficients obtained from this comprehensive multivariate analysis, as shown in the following formula:

$$\text{risk score}=\sum_{k=1}^{n}\text{coef}\left({lncRNA}^{i}\right)*expr\left({lncRNA}^{i}\right).$$

In this formula, coef(lncRNA) represents the multivariate Cox regression coefficient, expr(lncRNA) represents the expression level of lncRNA, and n represents the total number of lncRNAs to be added up. The risk scores of the samples were calculated, and the tumor samples were divided into high- and low-risk groups based on the median.

### 2.4 Substantiation of the risk model: concordance index and receiver operating characteristic

To assess the prediction ability of the risk model constructed, concordance index (C-index) and receiver operating characteristic (ROC) curves were used to evaluate the predictive accuracy of the risk model and multiple factors over time. The ROC curve visualizes a classifier’s prediction performance by plotting the true positive rate (TPR) against the false positive rate (FPR); the area under the curve (AUC) quantifies performance, with a higher AUC indicating better model accuracy. The ROC curves were plotted using the timeROC R package (Blanche et al., 2013). Similarly, the C-index measures the discriminatory power of a model. It calculates the proportion of all possible pairs of observations where the predicted order matches the actual outcome. A C-index of 1 indicates perfect prediction, while 0.5 suggests random prediction. The C-index was calculated using the R package rms (Harrell, 2022).

### 2.5 Construction of a nomogram, calibration curves, and decisive curve analysis

Multiple means were taken to further visualize the prediction ability of the risk model. A nomogram that incorporates the MPTRscore, age, and T and N stage to forecast the 1-year, 3-year, and 5-year OS using the rms package in R, which is a graphical tool that represents a statistical model allowing users to calculate probabilities or outcomes based on various input variables through a series of scales and lines, was constructed (Harrell, 2022). Additionally, calibration curves and decision curve analysis (DCA) capable of evaluating the predictive accuracy of the nomogram were produced. Calibration curves assess the agreement between predicted probabilities and observed outcomes. They plot predicted probability against the actual frequency of events, helping evaluate how well a model’s predictions align with reality using the rms R package (Harrell, 2022). In addition, DCA curves evaluate the clinical utility of a model by plotting the net benefit across different threshold probabilities using the rmda R package (Brown, 2018). They help compare models by assessing their effectiveness in decision-making.

### 2.6 Pathway enrichment analyses

Gene set variation analysis (GSVA) is a method used to estimate variations in gene set activity across samples. It transforms gene expression data into pathway-level scores, which facilitate the identification of biologically relevant changes, enhance the understanding of complex biological processes, and assess the impact of gene sets on phenotypic variations. On the other hand, gene set enrichment analysis (GSEA) identifies gene set enrichment by comparing ranked gene lists between groups. Using C2:KEGG gene sets, the SangerBox platform was utilized to perform GSEA to identify pathways significantly enriched between low- and high-risk groups (Shen et al., 2022). Pathways were considered significantly enriched based on a threshold of p < 0.05 and a |NES| greater than 1.5. Additionally, GSVA was directly applied to MPTRL using the platform LncSEA 2.0 (https://bio.liclab.net/LncSEA/index.php) with the dataset Experimental_Validated_Function, which includes pathological functions of lncRNAs validated through experiments (Chen et al., 2020).

### 2.7 Investigation of the TME and immune checkpoints

The expression levels of immune checkpoints and abundances of tumor-infiltrating immune cells can also be calculated from the gene expression profile. Based on the results of GSEA, the immune environmental milieu across different risk groups was examined. The immune infiltration statuses among BC patients were analyzed using CIBERSORT (Newman et al., 2015). The discrepancies in infiltrating immune cells were evaluated using the Wilcoxon signed-rank test and the ggpubr, reshape2, and ggplot2 R packages, with findings presented in a bar chart (Kassambara, 2023; Wickham, 2007; Wickham, 2016). Additionally, comparisons of TME scores and immune checkpoint activations between low- and high-risk groups were conducted using the ggpubr R package (Kassambara, 2023).

### 2.8 Therapeutic response prediction of the MPTRscore

The R package pRRophetic was employed to assess the response to chemotherapy, measured by the half-maximal inhibitory concentration (IC_50_) for each BC patient, based on data from the Genomics of Drug Sensitivity in Cancer (GDSC) (https://www.cancerrxgene.org/) (Geeleher et al., 2014). In addition, immunotherapeutic responses between the high and low MPTRscore groups were predicted using SubMap on the GenePattern (https://www.genepattern.org/) platform (Reich et al., 2006).

### 2.9 Identification of PAM50 classification subtypes

PAM50 is a 50-gene signature used to classify breast cancer into intrinsic subtypes, such as luminal A, luminal B, HER2, and basal-like subtypes. It serves as a gold-standard biomarker for BC, aiding in prognosis prediction and personalized treatment decisions based on tumor molecular characteristics. PAM50 classification subtypes were identified in the TCGA-BRCA cohort based on gene expression data using the R package genefu (Gendoo et al., 2016). The stratification of PAM50 subtypes enabled further analysis of the relationship between the MPTRscore and PAM50 classification subtypes.

### 2.10 Patient clustering and further analyses

Unsupervised consensus clustering is an automatic grouping method that aggregates results from multiple clustering algorithms to form a consensus. This approach enhances clustering stability and accuracy by identifying patterns consistent across different algorithms, providing a robust clustering solution. On the other hand, t-distributed stochastic neighbor embedding (t-SNE) and principal component analysis (PCA) are dimensionality reduction techniques that visualize high-dimensional data by mapping similarities into a lower-dimensional space to highlight clusters and patterns within the data. For the investigation of stratification in BC patients, potential molecular subtypes were identified using the ConsensusClusterPlus R package, with parameters set as follows: “distance” to Pearson, “clusterAlg” to pam, and “seed” to 123, based on the expression of five MPTRscore-related prognostic genes (Wilkerson and Hayes, 2010). The prognostic genes analyzed were selected by performing differential expression analysis with a threshold of p < 0.001 and logFC>0.585, along with univariate Cox regression analysis with a threshold of p < 0.05. In addition, the Rtsne R package was employed to conduct t-SNE analyses of immune function, while drug sensitivity analyses were conducted using the GSVA and pRRophetic R packages (Geeleher et al., 2014).

### 2.11 Single-cell analysis of expression distribution of MPTRscore-related prognostic genes in different cells in BC

Single-cell analysis investigates the unique molecular characteristics of individual cells, enabling a detailed exploration of gene expression and cellular heterogeneity. To characterize the gene expression patterns of MPTRscore-related clusters, the expression distribution of MPTRscore-related prognostic genes in different cells in BC was investigated on the platform ACLBI (https://www.aclbi.com/static/index.html#/) (Home for Reasearchers, 2025). The BRCA_GSE176078 single-cell data in the .h5 format and annotation results from TISCH were first downloaded, processed, and analyzed using R software MAESTRO and Seurat (Wang et al., 2020; Butler et al., 2018). Subsequently, cells were re-clustered using the t-SNE method. Finally, expression distributions of MPTRscore-related prognostic genes in different cells were analyzed.

### 2.12 Construction of an lncRNA–RNA interaction network linking MPTRLs and MPTRscore-related prognostic genes

To explore the molecular mechanism of the MPTRscore and the relationship between clusters and risk groups, an lncRNA–RNA interaction network was established. First, the co-expression network of MPTRLs and MPTRscore-related prognostic genes was constructed. It was built through Pearson correlation analysis between eight MPTRLs and five MPTRscore-related prognostic genes based on a threshold of p < 0.05 and |R| greater than 0.3. Furthermore, the RNA–RNA interactions between the selected RNAs were predicted based on the base-pairing principle and interaction energy with a threshold of −16 kcal/mol through the platforms LncRRIsearch (https://rtools.cbrc.jp/LncRRIsearch/index.cgi?t4=&hist=&em=em15) and ENCORI (https://rnasysu.com/encori/) (Fukunaga et al., 2019; Li et al., 2014; Zhou et al., 2025; Shannon et al., 2003).

### 2.13 Cell lines

Human normal mammary epithelial cells (MCF-10A) and breast cancer cells (SK-BR-3, MCF-7, HCC1806, BT549, MDA-MB-231, and Taxol-resistant MDA-MB-231) were purchased from the American Type Culture Collection (ATCC). The abovementioned cells were cultured in DMEM or RPMI 1640 medium (Gibco, CA, United States) supplemented with 10% FBS in a standard humidified incubator at 37°C with 5% CO_2_.

### 2.14 Quantitative real‐time polymerase chain reaction

Reverse transcription-quantitative polymerase chain reaction (RT-qPCR) is a technique used to measure RNA expression levels by converting RNA to cDNA. According to the manufacturer’s instructions, total RNAs were extracted from cells using TRNzol Universal Reagent (TIANGEN, Beijing, China), and complementary DNA synthesis was performed using a PrimeScript™ RT Reagent Kit with gDNA Eraser (TaKaRa, Kyoto, Japan). The expressions of RhoGDI2 and GAPDH were assessed using TB Green^®^ Premix Ex Taq™ II (TaKaRa, Kyoto, Japan). The relative expression of lncRNAs was analyzed using 2^−ΔΔCT^. The specific primer sequences used for target genes and reference genes (β-ACTIN) are listed in Table 1.

**TABLE 1:** Sequence of all primers.

Gene		Sequence
C9orf163	Forward primer	CCC​CAT​CTG​CTT​CTT​CCC​AG
	Reverse primer	CTC​TGC​ATC​CCC​CTC​TTT​GG
PSORS1C3	Forward primer	GAC​AGG​CCT​CGG​AAG​TCA​AA
	Reverse primer	CAC​TGG​GAG​ATG​AGG​TGC​TG
P11-23D24.2	Forward primer	CTA​CAG​GCT​TGG​TCA​GGA​TTT​G
	Reverse primer	TGT​AGG​GGT​ACT​CAG​GAC​TTT​G
P11-519C12.1	Forward primer	CCC​CTA​AGG​CTC​ATA​CAT​GGA
	Reverse primer	TTC​ACA​CAG​CAT​CCC​TCT​CA
P11-761I4.5	Forward primer	TGC​TTC​CAT​TCT​CCC​TGC​TC
	Reverse primer	ATG​GCC​CAT​CTC​TTC​CTT​GC
P5-1039K5.17	Forward primer	TAA​AAG​TGT​GGC​CCC​AGA​TCC
	Reverse primer	CCA​AAC​TGA​CGA​ACA​TCC​AGC
U47924.27	Forward primer	GGCCCGGTGACAGTAACC
	Reverse primer	CCC​ATT​GTT​CCC​CTT​TGC​CTA
USP30-AS1	Forward primer	TAC​GAC​GGT​TCC​CGA​GAC​A
	Reverse primer	TCC​GTC​AGC​TAT​TGC​TCT​CC
P11-573D15.8	Forward primer	AGC​AGA​GGA​AAA​GGA​TGG​GA
	Reverse primer	AAA​TGG​GAT​TGC​GAC​ACT​GC
β-ACTIN	Forward primer	TGA​CGT​GGA​CAT​CCG​CAA​AG
	Reverse primer	CTG​GAA​GGT​GGA​CAG​CGA​GG

### 2.15 siRNA transfection

The small interfering ribonucleic acid (siRNA) targeting the lncRNA transcript RP11-573D15.8 and control siRNA were bought from GenePharma (Shanghai, China). Following the manufacturer’s instructions, cells were transfected with GP-transfect-Mate (GenePharma, Shanghai, China). The transfected cells were collected for further studies after 48–72 h. The sequences of si-RP11-573D15.8 and si-NC were provided as follows:

siRP11-573D15.8 #1 sense strand: 5′-GGA​UUC​UAA​GUG​ACA​GAU​ACU-3′

antisense strand: 5′-UAU​CUG​UCA​CUU​AGA​AUC​CAA-3′

siRP11-573D15.8 #2 sense strand: 5′-GCU​GGG​UUA​UCC​AAA​CAU​AUU-3′

antisense strand: 5′-UAU​GUU​UGG​AUA​ACC​CAG​CAG-3′

siRP11-573D15.8 #3 sense strand: 5′-CCU​UGU​UCC​CAA​AGG​UUA​AAU-3′

antisense strand: 5′-UUA​ACC​UUU​GGG​AAC​AAG​GCA-3′

Negative control sense strand: 5′-UUC​UCC​GAA​CGU​GUC​ACG​UTT-3′

antisense strand: 5′-ACG​UGA​CAC​GUU​CGG​AGA​ATT-3′

### 2.16 Cell proliferation assays

Cells transfected with siRNAs were seeded on 96-well plates (1,000/well). Cell proliferation was detected using Cell Counting Kit-8 (APExBIO, Shanghai, China) after incubating with fresh medium for 0, 24, 48, 72, and 96 h. Thereafter, a 10% CCK-8 solution was added to each well and incubated for 2 h. Absorbance (OD) at 450 nm was determined using a Multiskan FC microplate reader (Thermo Fisher Scientific, Wilmington, United States).

### 2.17 Colony formation assays

For the colony-forming assay, cells transfected with siRNAs were seeded in six-well plates at a density of 1,000 cells per well. Then, cells were maintained in a medium containing 10% FBS (ExCell, Shanghai, China) for 14 days. Colonies were fixed with methanol, stained with crystal violet, and counted.

### 2.18 Transwell migration assays

For the transwell migration assay, the top 8-µm pore chambers (Millipore, United States) were seeded with 3 × 10^4^ MDA-MB-231 cells, and the lower chambers were filled with 600 μL DMEM medium (Gibco, CA, United States) with 10% FBS (ExCell, Shanghai, China). After 12 h, 4% paraformaldehyde was used to fix the migrated cells for 15 min, and then the cells were stained with crystal violet for 10 min and finally imaged under the microscope.

## 3 Results

### 3.1 Identifying MPT-related lncRNAs in BC patients

**FIGURE 1:**
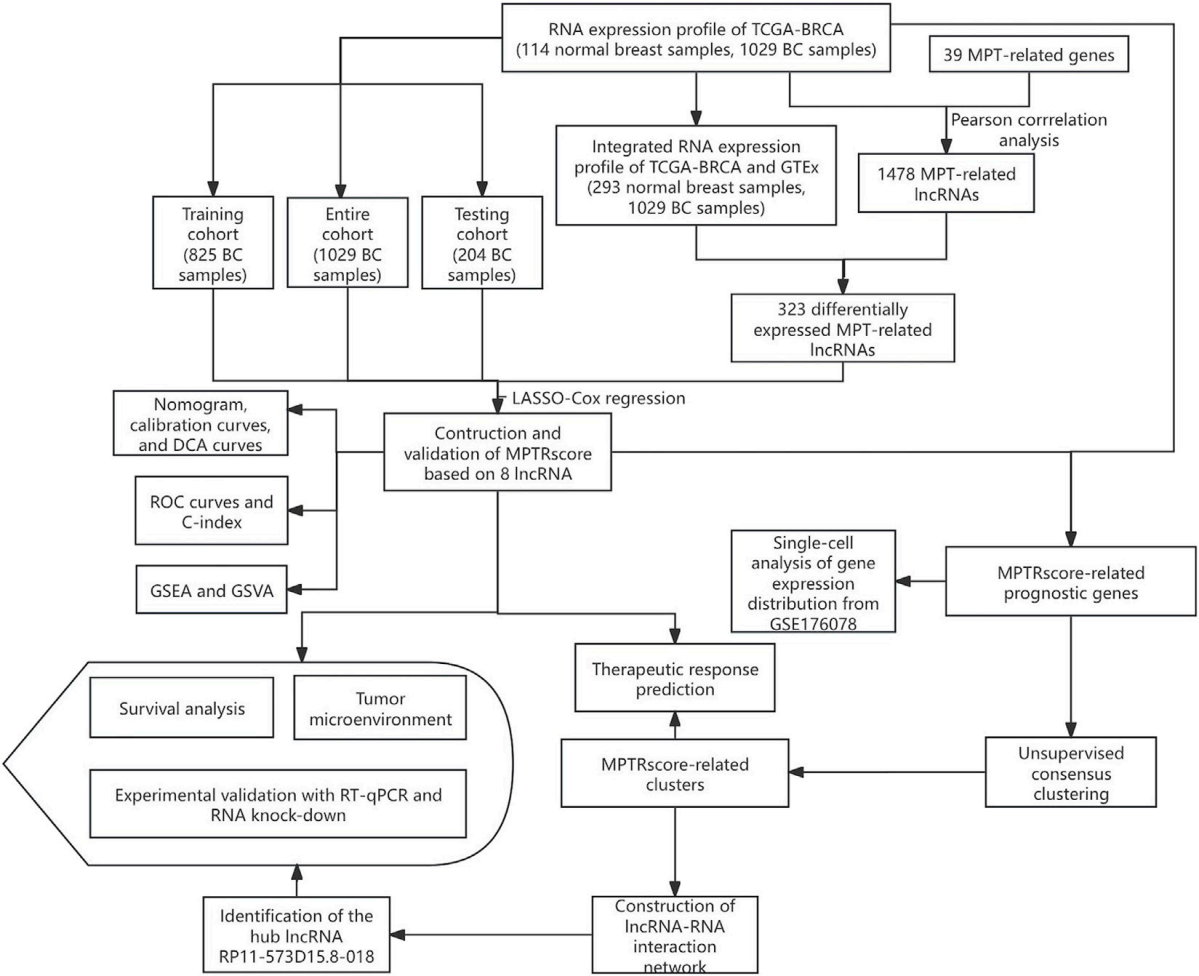
Schematic diagram showing the research design and the principal findings.

**FIGURE 2:**
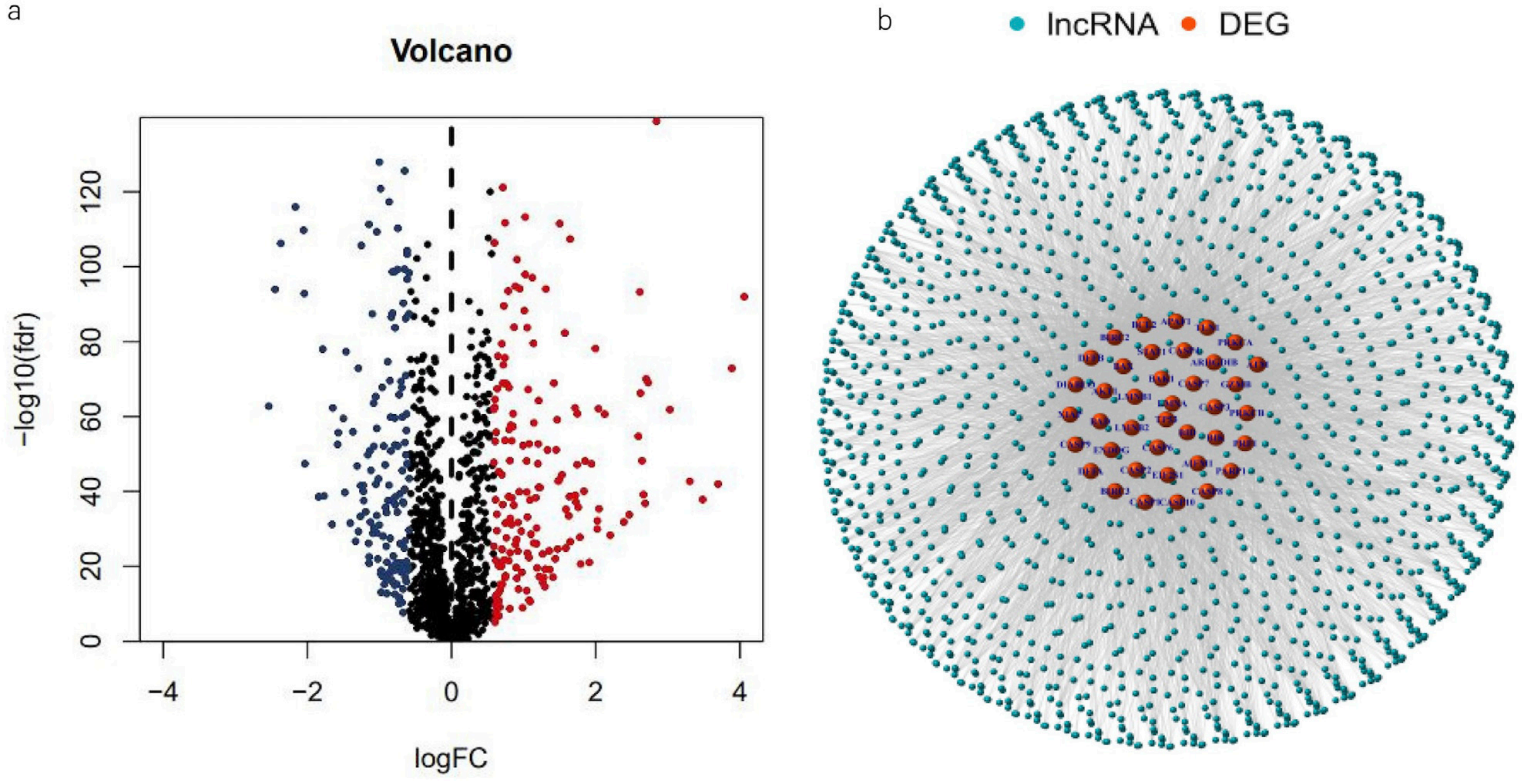
Spotting MPTRLs in BC patients. **(a)** Volcano plot representing 323 differentially expressed MPT-related genes. **(b)** Nnetwork analysis depicting the associations between MPT genes and lncRNAs, characterized by correlation coefficients greater than 0.3 and p-values less than 0.01.

The research design and key results are shown in Figure 1. A total of 98 normal and 1,029 tumor samples were collected from TCGA. Based on the expression profiles of 39 MPT-related genes and differentially expressed lncRNAs between the normal and tumor samples, 323 MPTRLs were identified. Among these, 150 were upregulated and the remaining were downregulated, as depicted in Figure 2A. Additionally, interactions between MPT-related genes, such as BLC2 and TP53, and lncRNAs are illustrated in Figure 2B.

### 3.2 Establishment and substantiation of the model

**FIGURE 3:**
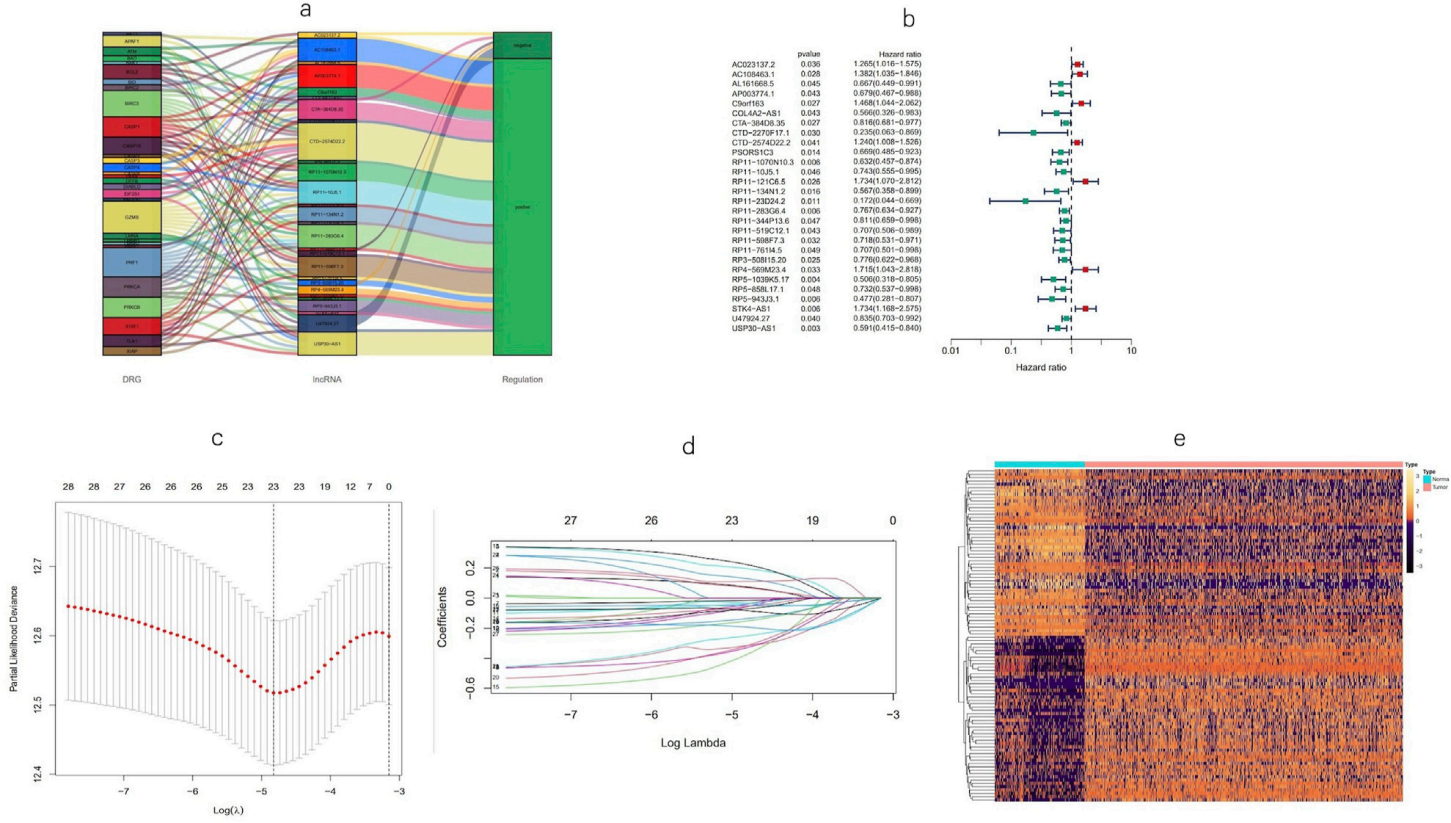
Identification of a prognostic signature for MPT-associated lncRNAs. **(a)** Forest plots depicting the screening of 28 lncRNAs using Cox regression in association with MPT. **(b)** Heatmap illustrating the expression patterns of the MPT-related lncRNAs between tumor and normal tissue. **(c, d)** LASSO regression analysis of the predictive model. **(e)** Sankey diagram representing lncRNAs co-expressed with genes related to MPT.

Using univariate Cox regression analysis, 28 MPT-related lncRNAs that were significantly correlated with OS (p < 0.05) were found (Figure 3B), and a heatmap (Figure 3E) was generated. To avoid overfitting the prognostic signature, LASSO regression was performed on these lncRNAs, and 23 MPTRLs were extracted in BC when the first-rank value of log(λ) corresponded to the minimum likelihood of deviance (Figures 3C, D). Among these 28 lncRNAs, 7 lncRNAs were upregulated, with the others being downregulated, as presented in the Sankey diagram (Figure 3A). Consequently, eight MPT-related lncRNAs independently and significantly correlated with OS (p < 0.05) were selected via multi-Cox regression. The risk score could be calculated using the following formula: risk score = C9orf163 × (0.442036850417968) + PSORS1C3 × (−0.31350517966782) + RP11-23D24.2 × (−1.11619026622422) + RP11-519C12.1 × (−0.43188554076601) + RP11-761I4.5 × (−0.46668668130283) + RP5-1039K5.17 × (−0.529899453401692) +U47924.27 × (−0.210875741139221) + USP30-AS1 × (−0.480922434407888).

**FIGURE 4:**
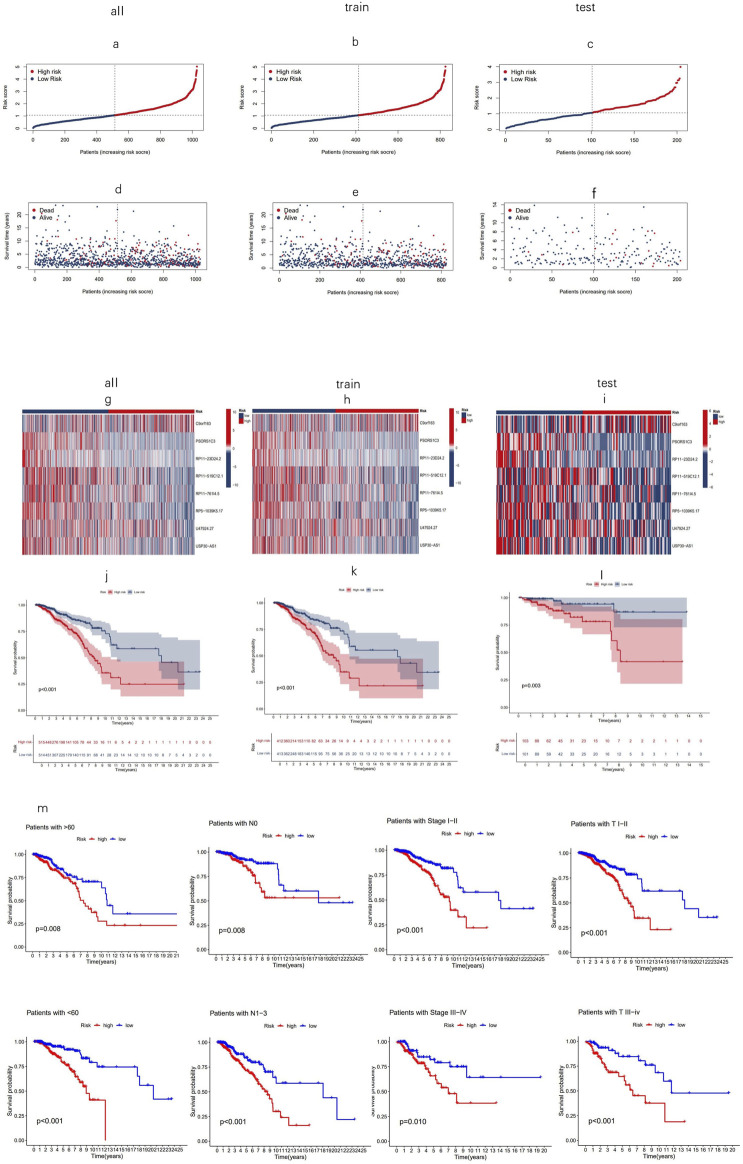
Prognostic value of eight MPT-related lncRNAs across training, testing, and entire cohorts. **(a–c)** Display of the MPT-related lncRNAs model based on risk scores for the training, testing, and entire cohorts. **(d–f)** Comparison of survival times and statuses between low- and high-risk groups in the training, testing, and entire cohorts. **(g–i)** Heatmaps showing the expression of eight lncRNAs in the training, testing, and entire cohorts. **(j–l)** Kaplan–Meier survival curves illustrating the OS of patients in low- versus high-risk groups across the training, testing, and entire cohorts. **(m)** Kaplan–Meier survival curves for OS stratified by age, stage, and T and N stage in high- and low-risk groups within the entire cohort.

Using the risk score formula, the distribution of risk score, the survival status, the survival time, and the relevant expression standards of these lncRNAs of patients were compared between low- and high-risk groups in the train, test, and entire sets. As shown in Figures 4A–I, patients with higher risk scores tended to have poorer overall survival. The Kaplan–Meier curves (Figures 4J–L) reinforce the prognostic power of this signature across training, testing, and entire cohorts. In addition, the conventional clinicopathologic characteristics, age, gender, grade, and T and N stages also indicated that these eight MPTRLs could serve as independent predictors of patient outcomes (Figure 4M).

### 3.3 Construction of a nomogram and the evaluation of the prognostic model

**FIGURE 5:**
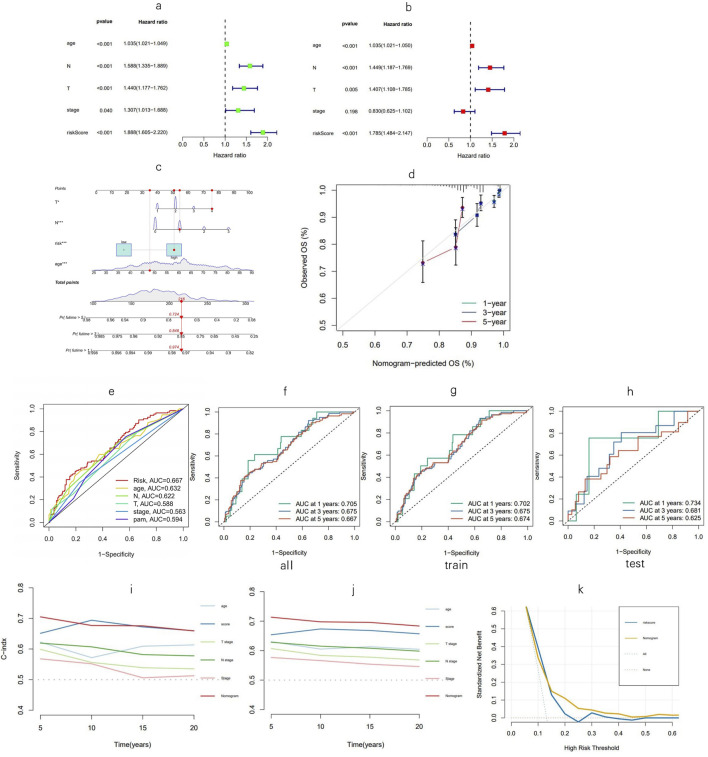
Nomogram evaluation and risk model assessment. **(a, b)** Univariate and multivariate Cox analyses assessing clinical factors and risk score in relation to OS. **(c)** Nomogram incorporating MPTRscore, age, and tumor stage to estimate the probabilities of 1-, 3-, and 5-year OS. **(d)** Calibration curves for predicting 1-, 3-, and 5-year OS outcomes. **(e)** Five-year ROC curves for evaluating the risk score, clinical factors, and PAM50 subtypes. **(f–h)** One-, three-, and five-year ROC curves for the training, testing, and entire cohorts, respectively. **(i, j)** C-index for the nomogram; validation of the C-index for the nomogram via the bootstrap method for evaluating the prognostic accuracy of the model and constitute variables. **(k)** DCA curves of the model and MPTRscore for evaluating their prognostic accuracy and net benefit.

Univariate Cox regression analysis indicated that age, T and N stages, tumor stage, and risk score, named MPTRscore, were prognostic risk factors for BC patients (Figure 5A). This initial finding underscores that the newly derived MPTRscore captures essential components of tumor aggressiveness and patient outcomes. Furthermore, multivariate Cox regression confirmed that age, T and N stages, and MPTRscore remained independent prognostic risk factors for BC patient outcomes (Figure 5B). This result highlights the unique contribution of the MPTRscore in predicting patient survival. Utilizing these four independent prognostic factors—MPTRscore, age, and T and N stages (all with p < 0.05 in the multivariate Cox analysis)—a nomogram was developed to predict 1-, 3-, and 5-year OS rates for BC patients (Figure 5C). Calibration plots for 1-, 3-, and 5-year predictions demonstrate that the nomogram closely aligns with actual outcomes, supporting its potential utility in a clinical setting (Figure 5D).

At the 5-year mark, ROC analysis of the risk score demonstrated superior prognosis prediction performance over other clinical factors in the TCGA-BRCA cohort, while the comparison with PAM50 subtypes as a gold standard biomarker contextualizes the prognosis prediction ability of the score (Figure 5E). Time-dependent ROC curves were used to assess the sensitivity and specificity of the model for prognosis, further confirming the high sensitivity and specificity of the model and reflecting its ability to capture dynamic changes in patient prognosis over time (Figures 5F–J). Furthermore, the time-dependent C-index of the nomogram was validated using bootstrap resampling 1,000 times. DCA curves of the MPTRscore and the risk model to evaluate the clinical prognosis benefit of biomarkers, demonstrated greater benefit for BC patients than the MPTRscore (Figures 5K).

### 3.4 GSEA, investigation of the association with the immune system and PAM subtype, and therapy sensitivity investigation

**FIGURE 6:**
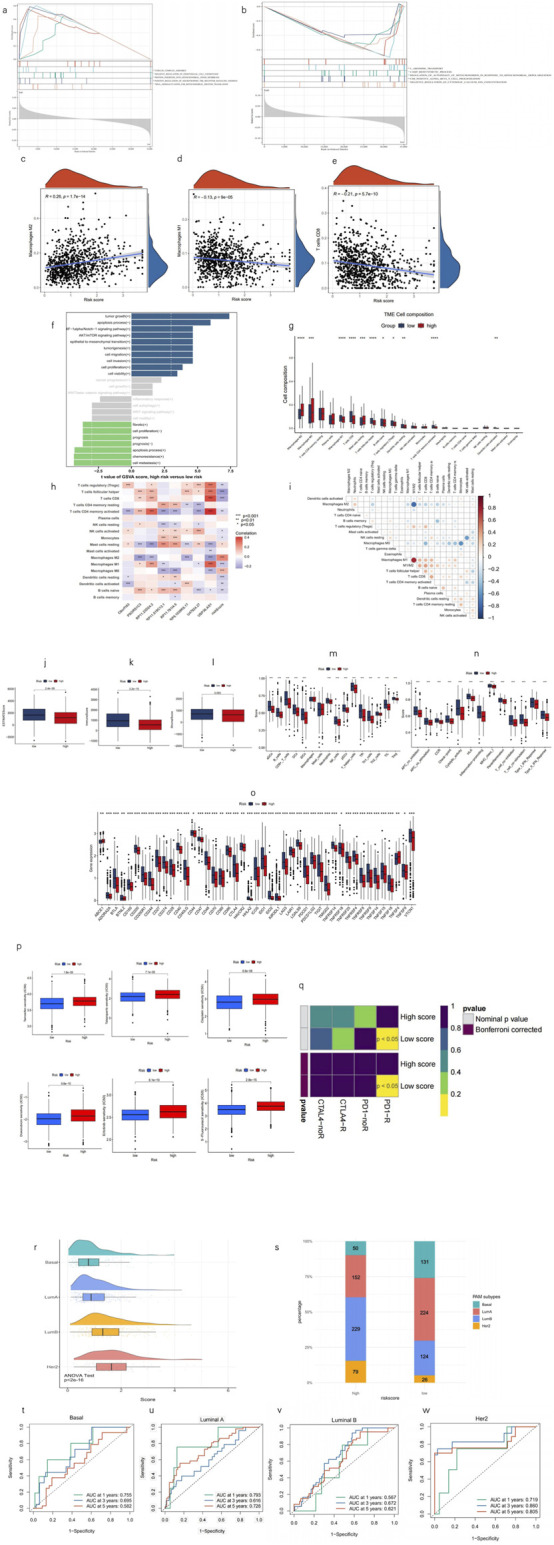
Analysis of tumor immune factors and therapy efficacy. **(a, b)** GSEA reveals five pathways that are upregulated, downregulated, and significantly enriched in the high-risk group, respectively. **(c–e)** Correlation analysis between the risk score and prevalence of M1, M2, and CD8 T cells. **(f)** GSVA via lncSEA demonstrates significant difference in pathological processes between risk groups. **(h)** Visualization of immune cell distributions across risk groups via a bar plot. *p < 0.05, **p < 0.01, ***p < 0.001, and ****p < 0.0001. **(g)** Heatmap shows the correlation of enrichment of immune cells with eight lncRNAs and MPTRscore. **(i)** Correlation between enrichment of TME infiltration immune cells. **(j–l)** Comparison of ESTIMATE results between high and low MPTRscore groups. **(m–o)** Differential expression of immune checkpoints and immune cells in the risk groups. *p < 0.05, **p < 0.01, and ***p < 0.001. **(p)** Predictive modeling of chemotherapy responsiveness in risk groups. **(q)** SubMap predicts immunotherapy sensitivity of high and low MPTRscore groups by comparing with a 47-sample immunotherapy melanoma cohort. **(r)** MPTRscore distribution of four PAM subtypes. **(s)** Distribution of four PAM50 classification subtypes in low- and high-risk groups. **(t–w)** ROC curves for predicting 1-, 3-, and 5-year OS of basal, luminal A, luminal B, HER2 subtypes of the TCGA cohort.

To assess variations in biological functions between risk groups, GSEA was employed to analyze the high-risk group’s enrichment in KEGG pathways across the entire dataset. Notably, the high-risk group was significantly enriched with the upregulation of MPT-promoting pathways and downregulation of MPT-suppressing pathways. Moreover, immune response pathways were also enriched in the high-risk group through the upregulation of endothelial cell chemotaxis and downregulation of CD8^+^ T cell proliferation, which indicates a poorer immune infiltration in TME. In addition, the high-risk group has a significant upregulation of the oncogenic signaling pathway of neurotrophin TRK receptor signaling, which has also been reported to elicit the intracellular calcium efflux that promotes MPT by increasing cytosolic calcium (Rose et al., 2003). Moreover, the upregulation of tubulin assembly in the high-risk group reveals a rapid cellular replication state in tumors. All of these pathways correspond to a worse prognosis (all p < 0.05; |NES| > 1.5) (Figures 6A, B). Following that, GSVA was performed using the LncSEA tool, in which the high-risk group was featured by significant upregulation of tumorigenic processes, metastasis, and a worse prognosis (Figure 6F).

A CIBERSORT-based analysis of TME immune infiltration was conducted within the MPTRscore framework. The results indicate that the low-risk group is more strongly associated with an inflammatory immune response, as reflected in the differential abundance of immune cell populations. The figure presents the composition of various immune cell types, highlighting statistically significant differences between the high- and low-risk groups (all p < 0.05) (Figure 6G). Furthermore, it was observed that lower risk scores were more closely related to anti-tumor immune TME, particularly high M1 and low M2 macrophages, and high CD8^+^ T-cell infiltration (Figures 6C–E). The correlation between the enrichment of TME infiltration immune cells also demonstrates that CD8^+^ T cells are positively correlated with M1 macrophage and negatively correlated with M2 macrophage. Moreover, M1/M2 even shows a stronger positive correlation to CD8^+^ T cells, indicating the existence of regulation between tumor-associated macrophage (TAM) and CD8^+^ T cells (Figure 6I). Furthermore, the correlation of each of the eight lncRNAs and TME immune cell infiltration is visualized in Figure 6H, in which USP30.AS1 and RP11.23D24.2 were two major contributors to the significant correlation between risk score and CD8^+^ T cells, M1 macrophage, and M2 macrophage. These findings indicate that the low-risk group is characterized by enhanced anti-tumor inflammatory immune infiltration.

Additionally, the high-risk group demonstrated a higher immune score and a higher ESTIMATE score, indicating that the TME had higher tumor purity and greater immune cell infiltration than the low-risk group (Figures 6J–L). Most immune cells and pro-inflammatory biological processes were also found to be more activated in the low-risk group (Figures 6M, N). Moreover, immune checkpoints in the low-risk group were mostly significantly lower than those in the high-risk group (Figure 6O). In addition, the TME infiltration of pro-inflammatory M2 macrophage was shown to be negatively correlated with CD8^+^ T cells. Meanwhile, as an immune cell responsible for immune response inhibition, the TME infiltration of M1 macrophage was positively correlated with CD8^+^ T cells, and this correlation was more pronounced for the M1/M2 ratio (Figure 6I). Additionally, the high-risk group, which had a higher MPTRscore, exhibited a higher IC_50_ value for chemotherapeutic drugs (Figure 6M). Moreover, analysis indicated that six chemotherapeutic drugs used in BC therapy had significantly lower IC_50_ values in the high-risk group, suggesting enhanced sensitivity to these treatments (Figure 6P). Additionally, responses to immunotherapy between the high- and low-risk groups were also analyzed, indicating that the low-risk group was significantly related to high sensitivity to anti-PD-1 immunotherapy (p < 0.05) (Figure 6Q). Additionally, relationship between MPTRscore and PAM50 classification subtypes was analyzed. As shown in Figure 6R, a significant difference was observed between the four PAM subtypes (p < 2e-16, ANOVA test), with MPTRscore increasing in order from basal-like, luminal A, luminal B, to HER2. Following that, significant differences in the distribution of the four PAM50 classification subtypes between the low- and high-risk groups were also demonstrated, as shown in Figure 6S. The basal-like and luminal A subtypes were more prevalent in the low-risk group, while the luminal B and HER2 subtypes were predominantly observed in the high-risk group. Finally, the prediction accuracy of the MPTRscore for OS in basal-like, luminal A, luminal B, and HER2 subtypes was calculated using ROC curves (Figures 6T–W). Overall, the MPTRscore demonstrated stable prediction accuracy spanning all the four subtypes, with the AUC of luminal A being the lowest and HER2 being the highest.

### 3.5 Identification and characterization of MPTRscore-related clusters

**FIGURE 7:**
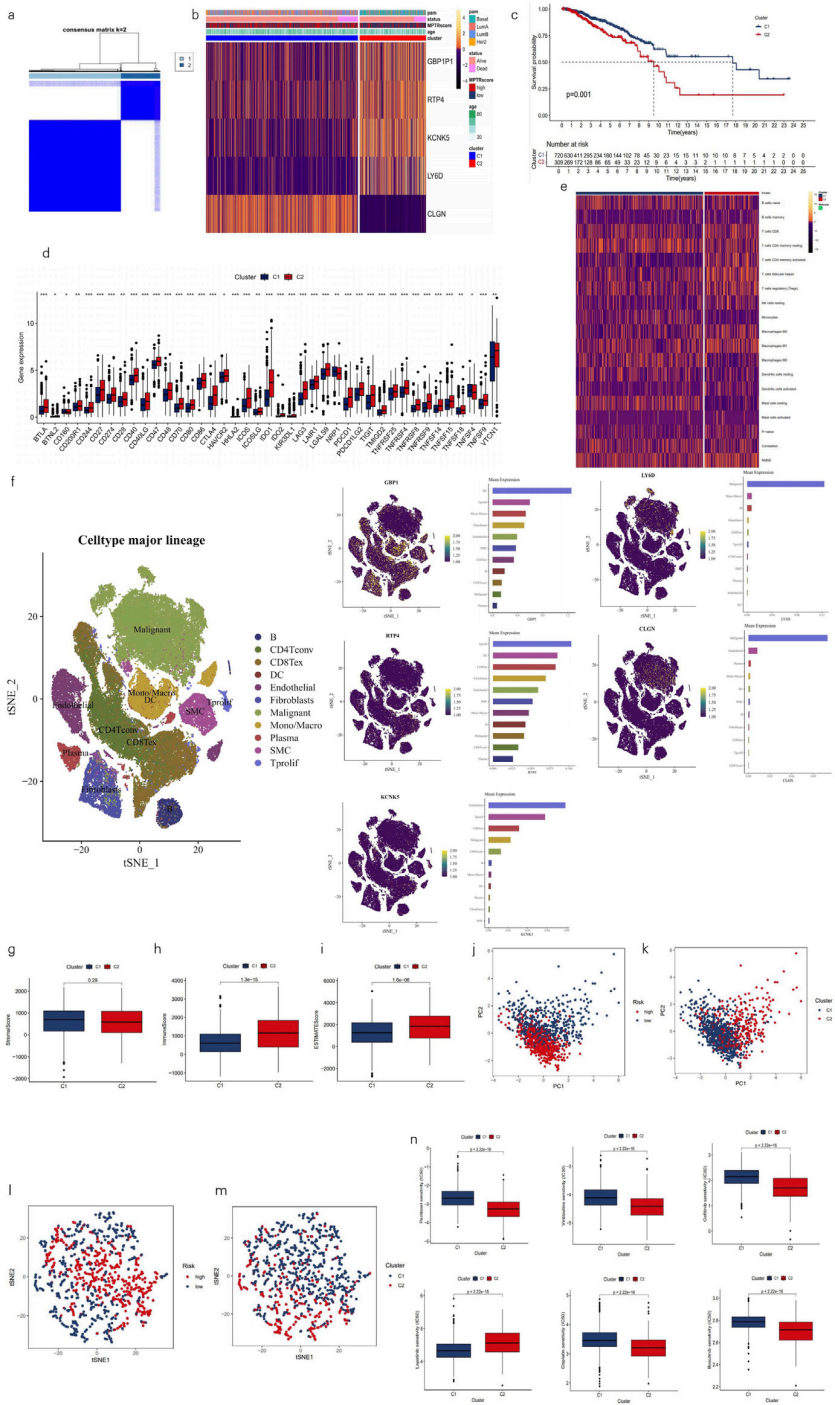
Characterization of two clusters based on MPTRscore-related prognostic gene expression. **(a)** Patients divided into two clusters in the ratio of 2:1 by unsupervised consensus clustering. **(b)** Heatmap illustrating the expression patterns of MPTRscore-related prognostic genes between clusters 1 and 2 with column annotations of clinical information and MPTRscore. **(c)** Kaplan–Meier survival curves of OS in clusters. **(d)** Difference of immune checkpoints expression in clusters. *p < 0.05, **p < 0.01, and ***p < 0.001 **(e)** Heatmap of immune cells in clusters. **(f)** t-SNE plot of single-cell sequencing analysis shows the expression distribution of GBP1, RTP4, KCNK5, and LY6D in different cells in the GSE176078 BRCA cohort. **(g–i)** Comparison of ESTIMATE results between clusters 1 and 2. **(j, k)** PCA of risk groups and clusters based on gene expressions. **(l, m)** t-SNE of two clusters based on gene expressions. **(n)** Prediction of chemotherapy responsiveness in clusters of the same six chemotherapy drugs.

Drawing on prior studies, it is recognized that breast cancer, as a highly heterogeneous tumor, can be divided into various clusters or subtypes, which typically exhibit distinct prognostic, biomolecular, and phenotypic characteristics. Utilizing differential expression analysis between high- and low-risk groups with a threshold of p < 0.001 and logFC>0.585, followed by univariate Cox regression with a threshold of p < 0.05 to select five MPTRscore-related genes (GBP1P1, RTP4, KCNK5, LY6D, and CLGN), samples were classified into two clusters using the ConsensusClusterPlus R package based on the expression patterns of these MPTRscore-related lncRNAs (Figures 7A, B) (Wilkerson and Hayes, 2010). Among the five MPTRscore-related genes, four are protein-coding genes, while GBP1P1 is a pseudogene. GBP1P1, RTP4, KCNK5, and LY6D were downregulated in cluster 2 and upregulated in cluster 1, which is the opposite of CLGN. Moreover, cluster 1 was more enriched with high MPTRscore and luminal A, luminal B, and HER2 subtypes, and cluster 2 was more enriched with low MPTRscore and basal-like subtypes (Figure 7B). To investigate the expression distribution of the five MPTRscore-related genes in different cells, single-cell sequencing analysis was performed by re-clustering cells using t-SNE on the GSE176078 BRCA cohort. As GBP1 acts as the counterpart targeting effector of the pseudogene GBP1P1, the analysis focused on the expression distribution of GBP1 rather than GBP1P1 (Mohebifar et al., 2023). GBP1, RTP4, and KCNK5 were found to be highly expressed in non-malignant cells, including T cells, macrophages, DCs, and endothelial cells. Moreover, LY6D and CLGN were found to be highly expressed in malignant cells (Figure 7F). Additionally, cluster 1 demonstrated significantly improved OS according to Kaplan–Meier analysis (Figure 7C). The majority of immune checkpoints demonstrated heightened expression in cluster 2 (Figure 7D). Moreover, cluster 2 exhibited higher levels of immune cell infiltration, as determined by CIBERSORT (Figure 7E). Cluster 2 exhibited elevated immune and ESTIMATE scores, indicating a TME with higher tumor purity and immune cell infiltration, similar to the high-risk group, compared to cluster 1 (Figures 7G–I). PCA was conducted to compare the group distributions between risk groups and clusters, showing the strong performance of five MPTRscore-related genes in capturing MPTRscore characteristics (Figures 7J, K). Additionally, t-SNE analysis also showed clear and similar distinctions between both clusters and risk groups (Figures 7L, M). Both dimensionality reduction analyses show the same correspondence between clusters and risk groups (cluster 1 aligning with the high-MPTRscore group and cluster 2 aligning with the low-MPTRscore group). Drug sensitivity analysis revealed that chemotherapeutic drugs exhibited the same trend of IC_50_ values across clusters and risk groups (Figure 7N).

### 3.6 Revelation of the linkage between two clusters and MPTRscore

**FIGURE 8:**
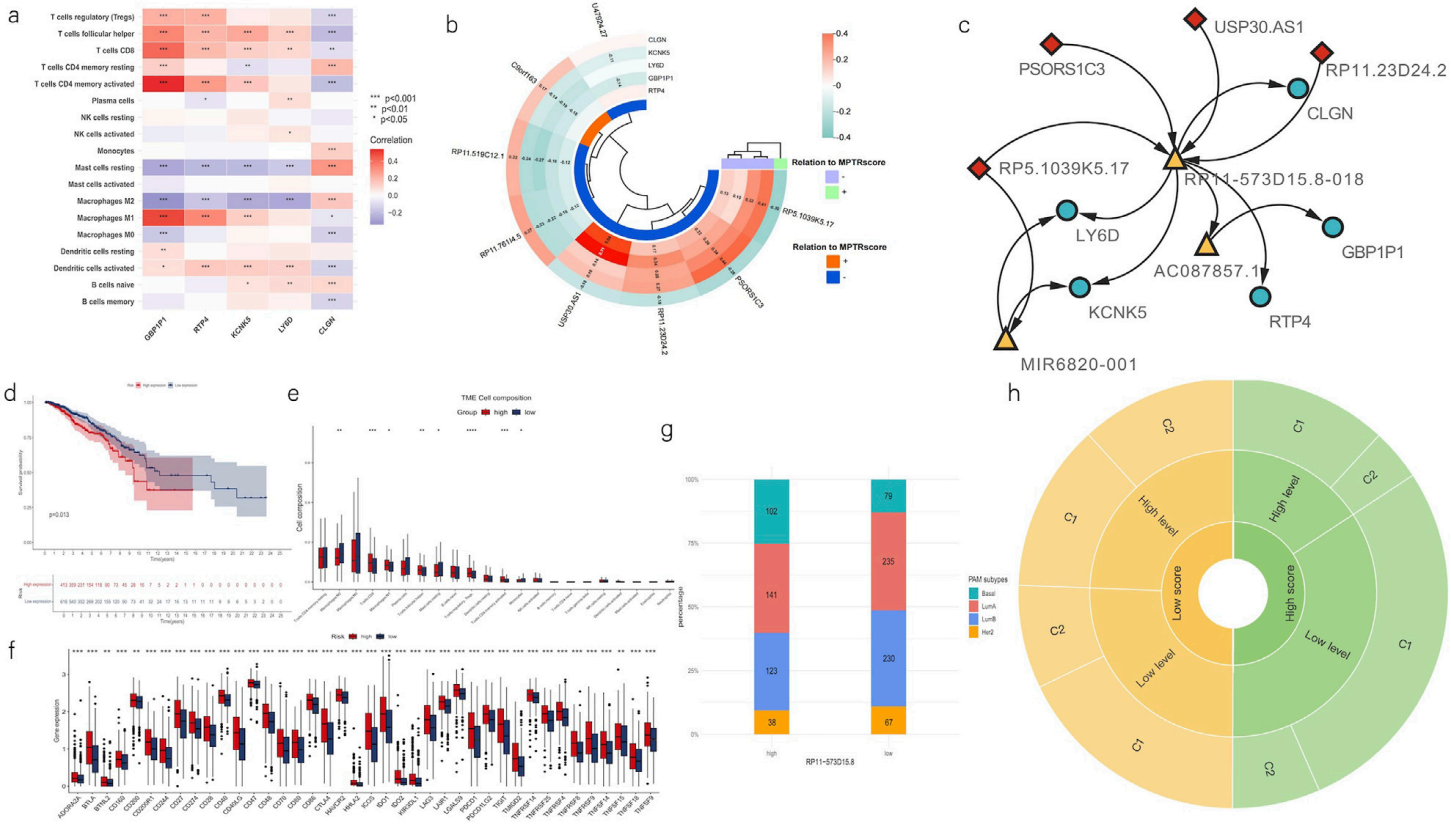
Linkage between two clusters and MPTRscore. **(a)** Heatmap shows the correlation of enrichment of immune cells with five MPTRscore-related prognostic genes. **(b)** Correlation between MPTRscore-related prognostic genes and MPTRLs. The values with p < 0.05 were hidden. **(c)** RNA–RNA interaction network shows the linkage between MPTRLs and MPTRscore-related prognostic genes. Arrow indicates the direction of interactions. MPTRLs are indicated in red lozenges, MPTRscore-related prognostic genes are shown in blue ellipses, and mediator RNAs are demonstrated in yellow triangles. **(d)** Kaplan–Meier survival curves of OS of high and low RP11-573D15.8 expression groups. **(e)** CIBERSORT estimates TME immune cell infiltrations across high and low RP11-573D15.8 expression groups. *p < 0.05, **p < 0.01, ***p < 0.001, and ****p < 0.0001. **(f)** Differential expression of immune checkpoints and immune cells between the high and low RP11-573D15.8 expression groups. **p < 0.01 and ***p < 0.001. **(g)** Distribution of four PAM50 classification subtypes in low and high RP11-573D15.8 expression groups. **(h)** Sunburst chart illustrating the association between MPTRscore, RP11-573D15.8 expression level, and MPTRscore-related prognostic clusters.

Based on the characteristics shared between the clusters and risk groups, deeper analyses were then performed to uncover the linkage between MPTRscore and clusters. First, the correlation between five MPTRscore-related prognostic genes and TME immune cell infiltration was also investigated. The results showed that GBP1P1, RTP4, and KCNK5 had a significant positive correlation to CD8 T cells and M1 macrophages, while showing a significant negative correlation with M2 macrophages (Figure 8A). Among these three genes, GBP1P1 demonstrated the strongest correlation. Afterward, the correlation between MPTRscore-related prognostic genes and MPTRLs was studied, revealing the strongest correlation between USP30.AS1 and GBP1P1 (Pearson correlation analysis, R^2^ = 0.71), followed by USP30.AS1 and RTP4 (Pearson correlation analysis, R^2^ = 0.54) (Figure 8B). Based on these findings, seven highly correlated pairs, including all five MPTRscore-related prognostic genes and four MPTRLs with higher correlation index (greater than 0.3), were selected for further investigation (USP30.AS1-GBP1P1, USP30.AS1-RTP4, RP11.23D24.2-GBP1P1, PSORS1C3-KCNK5, PSORS1C3-LY6D, RP5.1039K5.17-CLGN, and RP5.1039K5.17-KCNK5), and an RNA–RNA interaction network was then built using LncRRIsearch and ENCORI. The base-pairing information and interaction energies between RNAs are provided in the supplementary file. Consequently, a hub lncRNA transcript, RP11-573D15.8-018 (ENST00000627551), was identified as a mediator of the molecular linkage between all four selected MPTRLs and all five MPTRscore-related prognostic genes. Moreover, an alternative RNA–RNA interaction pathway was identified in RP5.1039K5.17-CLGN and RP5.1039K5.17-KCNK5, mediated by miRNA MIR6820-001 (Figure 8C).

Furthermore, to investigate the role of RP11-573D15.8-018 in linking two clusters and MPTRscore, the sum expression of the corresponding gene of the alternative splicing transcript RP11-573D15.8-018 was retrieved. RP11-573D15.8 was also one member of 323 MPTRLs that were formerly identified. First, Kaplan–Meier survival analysis of OS of high and low RP11-573D15.8 expression groups, with high and low RP11-573D15.8 expression groups divided in a ratio of 2:3, shows that a higher RP11-573D15.8 expression level corresponds to worse prognosis (p = 0.013, log-rank test) (Figure 8D). Then, CIBERSORT analysis was performed on high and low RP11-573D15.8 expression groups, displaying significantly higher CD8 T cell and M1 macrophage infiltration and lower M2 macrophage infiltration in the TME of the high RP11-573D15.8 expression group (Figure 8E). Moreover, immune checkpoints in the low RP11-573D15.8 expression group were significantly lower than those in the RP11-573D15.8 expression group (Figure 8F). Moreover, the high RP11-573D15.8 expression group was found to be enriched with basal-like PAM subtype, and the low RP11-573D15.8 expression group was found to be enriched with luminal and HER2 subtypes (Figure 8G). The same characteristics can be found in MPTRscore-related clusters. These analyses revealed that RP11-573D15.8 was strongly associated with MPTRscore-related clusters (with low RP11-573D15.8 expression corresponding to C1 and high RP11-573D15.8 expression corresponding to C2).

Finally, the association between MPTRscore, RP11-573D15.8 expression level, and MPTRscore-related prognostic clusters was illustrated using a sunburst chart (Figure 8H). High and low MPTRscore groups were evenly divided (1:1); high and low RP11-573D15.8 expression groups were divided at a 2:3 ratio; and clusters 1 and 2 were divided at a 2:1 ratio. Accordingly, a high MPTRscore is associated with low RP11-573D15.8 expression and cluster 1. A low MPTRscore is associated with high RP11-573D15.8 expression and cluster 2. Moreover, cluster 2 tends to accumulate at a high RP11-573D15.8 expression level. Cluster 1 tends to accumulate at a low RP11-573D15.8 expression level.

### 3.7 Expression of MPT-related lncRNAs *in vitro*

**FIGURE 9:**
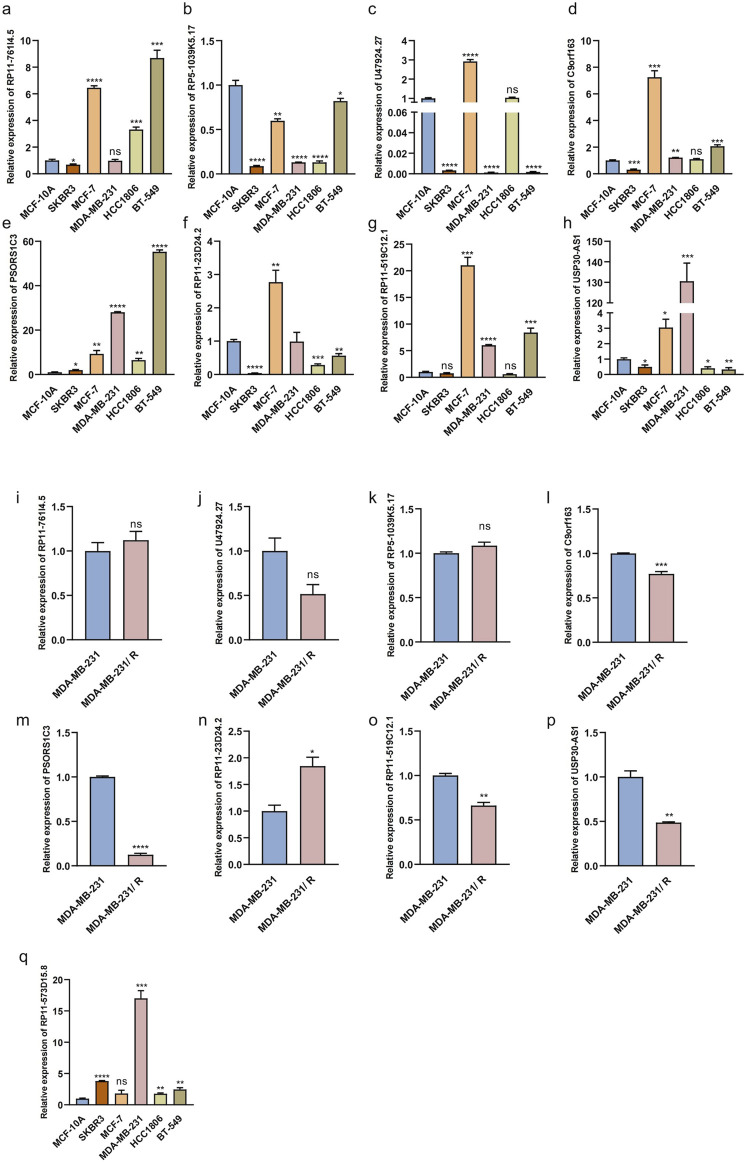
Relative expression of MPT-related lncRNAs in breast epithelial cell, breast cancer cell lines, and Taxol-resistant MDA-MB-231. **(a–h)** Relative expression levels of eight MPT-lncRNAs in breast epithelial cell MCF-10A, HER2-positive breast cancer cell line SKBR3, ER-positive breast cancer cell line MCF-7, and triple-negative breast cancer cell line (MDA-MB-231, HCC1806, and BT-549). **(i–p)** Relative expression levels of eight MPT-lncRNAs in Taxol-resistant MDA-MB-231 and MDA-MB-231. **(q)** Relative expression levels of RP11-573D15.8 in breast epithelial cell MCF-10A, breast cancer cell lines, and MDA-MB-231. Unpaired T-tests were used. *p < 0.05, **p < 0.01, ***p < 0.001, and ****p < 0.0001; ns, no significance.

We investigated the expression levels of the eight MPT-lncRNAs in different breast cancer cell lines and the MCF-10A noncancerous mammary epithelial line by RT-qPCR (Figures 9A–H). The results showed that all lncRNAs were downregulated in the HER2-positive breast cancer cell line SKBR3, except PSORS1C3 and RP11-519C12.1. In addition to RP5-1039K5.17, the lncRNAs were upregulated in the ER-positive breast cancer cell line MCF-7. However, the expression levels of the eight MPT-lncRNAs were not always consistent across three triple-negative breast cancer cell lines, namely, MDA-MB-231, HCC1806, and BT-549. In addition, we further investigated the expression levels of the eight MPT-lncRNAs of our model in Taxol-resistant MDA-MB-231 and MDA-MB-231 (Figures 9I–P). The results showed that RP11-23D24.2 was higher in Taxol-resistant MDA-MB-231 cells, and the other lncRNAs were lower or showed no significant difference. Finally, we examined the expression level of RP11-573D15.8 in MCF-10A and breast cancer cell lines (Figures 9Q). The results showed that the expression of RP11-573D15.8 was upregulated in SKBR3, MDA-MB-231, HCC1806, and BT-549, except in the ER-positive breast cancer cell line MCF-7.

### 3.8 Silencing RP11-573D15.8 suppressed the proliferation and migration in BC cells

**FIGURE 10:**
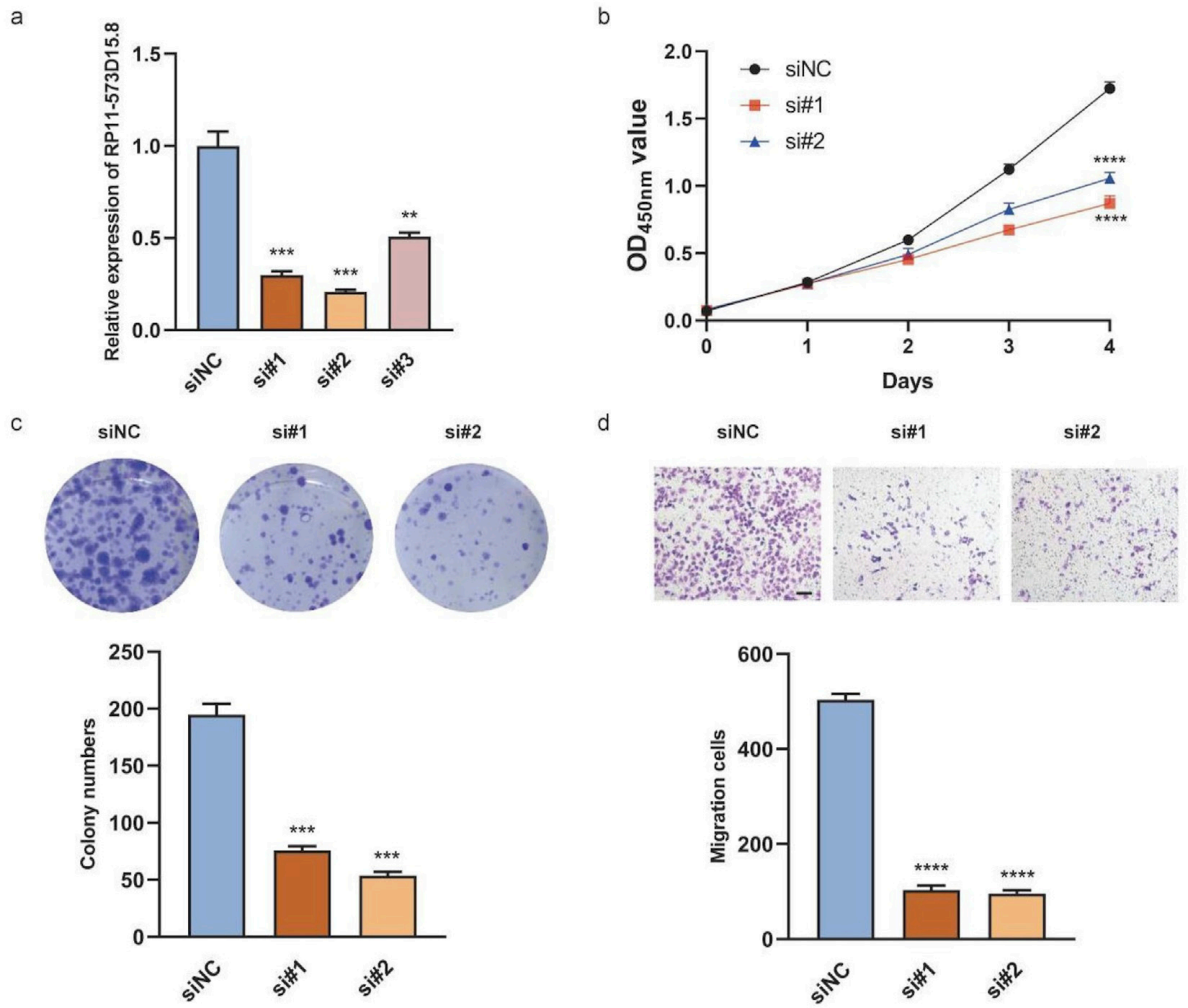
RP11-573D15.8 promotes cell proliferation and migration in MDA-MB-231 cells. **(A)** Relative qRT-PCR analysis for the validation of lncRNA transcript RP11-573D15.8 knockdown in MDA-MB-231 cells. **(B)** Effects of RP11-573D15.8 knockdown by siRNA on cell growth were measured using the CCK-8 assay in MDA-MB-231 cells. **(C)** Effects of RP11-573D15.8 knockdown by siRNA on cell growth were measured by colony formation in MDA-MB-231 cells. **(D)** RP11-573D15.8 knockdown by siRNA blocked the migration of MDA-MB-231 cells. Scale bar, 100 μm. Unpaired t-tests were used. *p < 0.05, **p < 0.01, ***p < 0.001, and ****p < 0.0001; ns, no significance.

To investigate the biological functions of the hub lncRNA transcript RP11-573D15.8 in breast cancer, we transfected MDA-MB-231 cells with siRNA to inhibit the endogenous expression of RP11-573D15.8 since the expression of RP11-573D15.8 in MDA-MB-231 cells was nearly twenty times higher than that in MCF-10A cells (Figure 9Q). The qRT-PCR results revealed that the expression of RP11-573D15.8 was successfully decreased in MDA-MB-231 using sequences 1 and 2 (Figure 10A). Cell proliferation and colony formation assays showed that the downregulation of RP11-573D15.8 could significantly reduce the proliferation ability of MDA-MB-231(Figures 10B, C). In addition, we performed a transwell assay to evaluate the effect of RP11-573D15.8 on cell migration and found that the inhibition expression of RP11-573D15.8 significantly impaired the migration ability of MDA-MB-231 cells (Figure 10D).

## 4 Discussion

BC is a malignancy that presents several challenges in both therapy and prognosis due to its heterogeneity and diverse subtypes. Clinically, BC prognosis has traditionally relied on TNM and PAM50 classifications that provide a framework for assessing disease severity and survival probabilities (Kasangian et al., 2017; Wallden et al., 2015). Moreover, the implementation of molecular profiling, such as the 21-gene recurrence score assay (Oncotype DX), has quantified the risk of recurrence in early-stage, hormone-receptor-positive BC (Sparano et al., 2018). However, the subpopulations of BC remain incompletely characterized, highlighting the urgent need for reliable prognostic biomarkers for BC.

MPT has been indicated to be associated with certain mechanisms of the regulation of cancer cell death. MPT can be highly associated with the mPTP, which plays a pivotal role in regulating cell fate, especially in the case of cancer. Research has highlighted that the mPTP facilitates a critical balance between cell survival and death, with its opening being a key event in initiating cell death pathways (Bonora and Pinton, 2014). In BC, apoptosis in BC cells resulting from MPT was supported by a novel mPTP-dependent mechanism that works mainly through ROS surge (NavaneethaKrishnan et al., 2018). MPT-driven necrosis is a form of cell death that can play a significant role in the progression of various cancers (Galluzzi et al., 2018). Even if MPT has not been widely studied regarding the aspect of its interplay with either cancer therapy or cancer treatment, certain studies have suggested the availability of MPT-driven necrosis as a novel therapeutic target for cancer and the intricate function that MPT-driven necrosis possesses in the TME (Yu et al., 2020; Rodriguez-Ruiz et al., 2020). Even though few evidence suggests that MPT can be cohesively associated with BC through lncRNAs, there is abundant evidence that lncRNAs are highly related to BC (Zhu et al., 2021; Mozdarani et al., 2020). Hence, the MPT-related system was developed. Based on this scoring system, a clinical prediction model was then constructed by incorporating the clinical parameters, including age and T and N stages, which displayed moderate accuracy in predicting prognosis and therapeutic response.

Using LASSO-Cox regression, a scoring system called the MPTRscore was developed to assess individuals with breast cancer, considering individual differences. It was found that the low MPTRscore group had increased immune activation, while the high MPTRscore group showed immune suppression, suggesting that the MPTRscore could predict immunotherapy response. More importantly, the MPTRscore was identified as a TAM (tumor-associated macrophage) and CD8^+^ T cell-related score, which indicates the significant roles of MPTRLs in tumor regulation. Interestingly, the permeability of mitochondria influences mitochondrial DNA (mtDNA), which is pivotal in determining the roles and characteristics of TAMs within the TME. Dysfunctional mitochondria under oxidative stress can release mtDNA, significantly affecting TAM behavior by enhancing their immunosuppressive and tumor-promoting functions. Furthermore, the oxidation of mtDNA has been linked to the inhibition of CD8^+^ T-cell activation. This connection underscores a potential relationship between MPTRL and mtDNA, exploring which could yield new insights into the treatment and prognosis of breast cancer (Cheng et al., 2020; Guo et al., 2022). In conclusion, the investigation of the MPTRscore demonstrated its ability to distinguish immune patterns in BC. This was supported by GSEA, indicating that the MPTRscore was inversely correlated with the immunotherapy response. Hence, the response to immunotherapy involves various factors such as immune cell infiltration in the TME and intracellular processes. These findings imply that the MPTRscore could influence these key parameters and potentially predict the effectiveness of both immunotherapy and chemotherapy.

The stratification of BC was simultaneously achieved by identifying two clusters based on unsupervised consensus clustering with five MPTRscore-related prognostic genes. These five MPTRscore-related prognostic genes effectively capture the gene expression characteristics of high and low MPTRscore groups, as demonstrated by dimensionality reduction analyses (cluster 1 corresponds to the high MPTRscore group; cluster 2 corresponds to the low MPTRscore group). These two clusters also present significant differences in survival advantages (cluster 1 corresponds to a better prognosis; cluster 2 corresponds to a worse prognosis) and TME immune cell infiltration, immune checkpoint expressions, and chemotherapy sensitivity. Moreover, lower drug sensitivity is found in the high-risk group, with a significant difference (p < 2.22 × 10^−16^) in response to chemotherapy. Therefore, clusters 1 and 2, respectively, closely resemble the high and low MPTRscore groups in multiple dimensions, including TME immune cell infiltration, chemotherapy sensitivity, and gene expression pattern. However, a few signatures, including prognosis and immune checkpoint expressions, were inverted between clusters and MPTRscore groups. The inverted immune checkpoint expressions can lead to inverted immune anti-tumor activity and then contribute to the inverted prognosis. This indicates that the stratification based on the five MPTRscore-related prognostic genes does not capture the complete characteristics of the MPTRscore. It mainly captures the signatures of the MPTRscore in TME immune cell infiltration and chemotherapy sensitivity but not completely in the immune checkpoint expressions. Nevertheless, all these indicate the robust capacity of BC subtyping of the MPTRscore.

MPTRscore is also a PAM50-classification-subtype-related score. A higher prevalence of luminal B and HER2 subtypes was observed in the high-risk group, while the low-risk group was enriched with luminal A and basal-like subtypes. In addition, this was also supported by the same distribution in MPTRscore-related clusters. Luminal A subtype is typically associated with a favorable prognosis due to its lower proliferation rate and higher expression of hormone receptors than luminal B, which exhibits higher proliferation and a slightly worse prognosis despite also being hormone receptor-positive (Parker et al., 2009). In contrast, the HER2 subtype is characterized by the overexpression of the HER2 gene, correlating with a more aggressive disease course and a poorer prognosis than that of the luminal subtypes, though it may respond well to HER2-targeted therapies (Slamon et al., 1989). However, there is a paradox that the basal-like subtype is actually the most aggressive type of BC in the PAM50 subtyping system but is more accumulated in the low MPTRscore group. Since it has been demonstrated that MPTRL can be highly associated with TAMs and CD8^+^ T cells, one possible explanation is the interruption of the immune microenvironment: basal-like BC cells have been indicated to be significantly associated with a reduced risk of metastasis with higher infiltration of CD8^+^ T cells, and the presence of M2 macrophages in the basal-like subtype BC is associated with an increased risk of metastasis, which was found from Cox regression analyses that assessed the relationship between various immune gene sets and metastasis-free survival across different PAM50 BC subtypes (Hammerl et al., 2020). Although the specific mechanism is elusive, further prognostic significance of the relationship between MPTRLs and the immune microenvironment of BC is highlighted. Therefore, further studies are recommended to demonstrate the specific relationship between MPTRL, CD 8 + T cells, TAM, and basal-like BC cells.

As a necrosis-driven factor, MPT is typically regarded as a tumor-suppressing factor. Interestingly, a paradox was found according to GSEA that the high MPTRscore group is related to the downregulation of MPT and the low-risk group is related to the activation of MPT. Moreover, a similar paradox was also discovered in MPTRscore-related clusters. For the five MPTRscore-related prognostic genes, only CLGN is upregulated in cluster 1 and downregulated in cluster 2. Endoplasmic reticulum chaperone calmegin (CLGN) plays a pivotal role in regulating the intracellular calcium ion level on the endoplasmic reticulum membrane to avoid excessive calcium ions in the cytosol (Itcho et al., 2020). Because MPT is a calcium-dependent mitochondrial inner membrane transition, upregulated CLGN corresponds to higher MPT activation in cluster 1 (Bernardi et al., 2023). Single-cell sequencing analysis validated that CLGN is mainly expressed in malignant cells, which implies that increased MPT in malignant cells is associated with a better prognosis in cluster 1. The other four MPTRscore-related prognostic genes downregulated in cluster 1 have not been reported to be positively related to MPT, but they still serve as biomarkers for BC, corresponding to a worse prognosis (Mohebifar et al., 2023; Mayama et al., 2018; Alvarez-Baron et al., 2011; Fuchs et al., 2013). From the view of single-cell sequencing analysis, GBP1, RTP4, and KCNK4 all show low expression levels in malignant cells but high expression in T cells, macrophages, DC, and endothelial cells, which indicates a suppressing function in some tumor-suppressing cells. Therefore, a feasible hypothesis is that the MPTRscore is mainly influenced by tumor-suppressing cells rather than tumor malignant cells in the TME, due to the cellular heterogeneity of bulk RNA-seq. The results of single-cell sequencing analysis of three MPTRscore-related prognostic genes, demonstrating high MPTRscore-related prognostic gene expression in tumor-suppressing cells, partially support this hypothesis. This hypothesis accounts for the MPTRscore to some extent in the view of cellular heterogeneity, but further research is also needed.

The molecular mechanism linkage bridging MPTRscore and MPTRscore-related clusters was successfully identified. The five MPTRscore-related prognostic genes capture the characteristics of four MPTRLs (PSORS1C3, USP30.AS1, RP11.23D24.2, and RP5.1039K5.17) within the MPTRscore. The linkage is largely mediated by the lncRNA transcript RP11-573D15.8-018 through an RNA–RNA interaction mechanism. These four MPTRLs bind and sequester RP11-573D15.8-018, reducing its availability to regulate downstream lncRNAs. As validation, the expression level of the RP11-573D15.8 gene effectively distinguished MPTRscore-related clusters, indicating that RP11-573D15.8-018 could serve an ideal biomarker for recognizing these clusters. Additionally, two bypassing ways mediated by miRNA MIR6820-001 were also identified (RP5.1039K5.17-MIR6820-001-CLGN and RP5.1039K5.17-MIR6820-001-KCNK5). The lncRNA RP5.1039K5.17 can also influence the expression level of protein-coding mRNAs CLGN and KCNK5 by sequestering the mediator miRNA MIR6820-001. This mechanism is in accordance with the hypothesis of the endogenous RNA (ceRNA) network, proposing that ncRNAs, including lncRNA, influence downstream mRNA transcription by acting as sponges for mRNA-targeting microRNAs (miRNAs) (Yang et al., 2023). Therefore, two ceRNA axes were also found in this study. Meanwhile, RP11-573D15.8-018 plays the most central role in linking MPTRscore and MPTRscore-related clusters overall. Finally, through the abovementioned analyses, correspondence among the three levels was uncovered: high MPTRscore–low RP11-573D15.8 expression level–cluster 1 and low MPTRscore–high RP11-573D15.8 expression level–cluster 2.

Some limitations still exist in this study. First, in the initial step of filtering candidate MPTRLs, the Pearson correlation-based approach alone may not have effectively reduced confounding factors. Despite that, in the following steps, more effective methods were employed to mitigate confounding effects. For instance, the multivariate Cox regression incorporates key clinical potential confounding covariants such as age, T stage, N stage, and overall stage. By doing so, these prognostic factors can be controlled and the prediction independence of the MPTRscore can be ensured. In addition, the MPTRLs determined in this study are based solely on the statistic correlations of RNA expression levels. Given that the association between MPT and lncRNAs has not been widely studied, the statistic relationships do not definitively establish a regulatory relationship. Therefore, experiments to identify the lncRNAs among MPTRLs affecting MPT in BC cells should be conducted to eventually validate this relationship. Moreover, to further reduce bias produced in statistical analyses, cohorts with larger sample sizes are required, and the baseline of datasets needs to be controlled. Furthermore, considering the high cellular heterogeneity of BC, the gene expressions of eight MPTRLs and MPTRscore could be investigated through single-cell sequencing analysis to identify the contributions of each cell cluster in BC tumors to the MPTRscore. Additionally, as an alternative splicing transcript, transcribed from the gene RP11-573D15.8, RP11-573D15.8-018 only accounts for a partial percentage of the total RP11-573D15.8 expression. To better understand its specific functions from a transcriptomic perspective, its individual expression value should be extracted from the total RP11-573D15.8 expression through sequence alignment. Moreover, the predicted molecular mechanisms of RNA–RNA interactions still need experimental validation in the future.

In this study, the MPTRscore effectively stratifies breast cancer patients and is associated with prognosis, immune cell infiltration in the TME, and immune and molecular characteristics. Additionally, the MPTRscore could serve as a standalone prognostic tool for breast cancer patients and aid in guiding decisions regarding immunotherapy and chemotherapy. Additionally, RP11-573D15.8-018 (ENST00000627551) plays a central role in the molecular mechanism of the MPTRscore, and it can also act as a single biomarker in BC stratification. More importantly, the findings provide valuable insights into the potential application of MPT-related lncRNAs in breast cancer treatment.

## Data Availability

The datasets presented in this study can be found in online repositories. The data presented in the study are deposited in the repository Zenodo, accession DOI: https://doi.org/10.5281/zenodo.13118445.

## References

[B1] Alvarez-Baron C. P. Jonsson P. Thomas C. Dryer S. E. Williams C. (2011). The two-pore domain potassium channel KCNK5: induction by estrogen receptor α and role in proliferation of breast cancer cells. Mol. Endocrinol. 25 (8), 1326–1336. 10.1210/me.2011-0045 21680658 PMC3146246

[B2] Bauer T. M. Murphy E. (2020). Role of mitochondrial calcium and the permeability transition pore in regulating cell death. Circulation Res. 126 (2), 280–293. 10.1161/CIRCRESAHA.119.316306 31944918 PMC8317591

[B3] Bernardi P. Gerle C. Halestrap A. P. Jonas E. A. Karch J. Mnatsakanyan N. (2023). Identity, structure, and function of the mitochondrial permeability transition pore: controversies, consensus, recent advances, and future directions. Cell Death and Differ. 30 (8), 1869–1885. 10.1038/s41418-023-01187-0 PMC10406888 37460667

[B4] Bhan A. Soleimani M. Mandal S. S. (2017). Long noncoding RNA and cancer: a new paradigm. Cancer Res. 77 (15), 3965–3981. 10.1158/0008-5472.CAN-16-2634 28701486 PMC8330958

[B5] Blanche P. Dartigues J.-F. Jacqmin-Gadda H. (2013). Estimating and comparing time-dependent areas under receiver operating characteristic curves for censored event times with competing risks. Stat. Med. 32, 5381–5397. 10.1002/sim.5958 24027076

[B6] Bonora M. Giorgi C. Pinton P. (2022). Molecular mechanisms and consequences of mitochondrial permeability transition. Nat. Rev. Mol. cell Biol. 23 (4), 266–285. 10.1038/s41580-021-00433-y 34880425

[B7] Bonora M. Pinton P. (2014). The mitochondrial permeability transition pore and cancer: molecular mechanisms involved in cell death. Front. Oncol. 4, 302. 10.3389/fonc.2014.00302 25478322 PMC4235083

[B9] Butler A. Hoffman P. Smibert P. Papalexi E. Satija R. (2018). Integrating single-cell transcriptomic data across different conditions, technologies, and species. Nat. Biotechnol. 36 (5), 411–420. 10.1038/nbt.4096 29608179 PMC6700744

[B10] Chen J. Zhang J. Gao Y. Li Y. Feng C. Song C. (2020). LncSEA: a platform for long non-coding RNA related sets and enrichment analysis. Nucleic Acids Res. 12, D969–D980. 10.1093/nar/gkaa806 PMC7778898 33045741

[B11] Cheng A. N. Cheng L.-C. Kuo C.-L. Lo Y. K. Chou H.-Y. Chen C.-H. (2020). Mitochondrial Lon-induced mtDNA leakage contributes to PD-L1–mediated immunoescape via STING-IFN signaling and extracellular vesicles. J. Immunother. cancer 8 (2), e001372. 10.1136/jitc-2020-001372 33268351 PMC7713199

[B12] Colaprico A. Silva T. C. Olsen C. Garofano L. Cava C. Garolini D. (2015). TCGAbiolinks: an R/Bioconductor package for integrative analysis of TCGA data. Nucleic Acids Res. 44, e71. 10.1093/nar/gkv1507 26704973 PMC4856967

[B13] Dalla Via L. N Garcia-Argaez A. Martinez-Vazquez M. Grancara S. Martinis P. Toninello A. (2014). Mitochondrial permeability transition as target of anticancer drugs. Curr. Pharm. Des. 20 (2), 223–244. 10.2174/13816128113199990033 23701547

[B14] Fuchs E. Huber J. Pelletier A. Tarek H. Park M. Fukui Y. (2013). Rac-specific guanine nucleotide exchange factor DOCK1 is a critical regulator of HER2-mediated breast cancer metastasis. Proc. Natl. Acad. Sci. U. S. A. 110 (18), 7434–7439. 10.1073/pnas.1213050110 23592719 PMC3645577

[B15] Fukunaga T. Iwakiri J. Ono Y. Hamada M. (2019). LncRRIsearch: a web server for lncRNA-RNA interaction prediction integrated with tissue-specific expression and subcellular localization data. Front. Genet. 10, 10. 10.3389/fgene.2019.00462 31191601 PMC6546843

[B16] Galluzzi L. Vitale I. Aaronson S. A. Abrams J. M. Adam D. Agostinis P. (2018). Molecular mechanisms of cell death: recommendations of the nomenclature committee on cell death 2018. Cell Death and Differ. 25 (3), 486–541. 10.1038/s41418-017-0012-4 PMC5864239 29362479

[B17] Geeleher P. Cox N. Huang R. S. (2014). pRRophetic: an R package for prediction of clinical chemotherapeutic response from tumor gene expression levels. PLoS One 9 (9), e107468. 10.1371/journal.pone.0107468 25229481 PMC4167990

[B18] Gendoo D. M. Ratanasirigulchai N. Schroder M. S. Pare L. Parker J. S. Prat A. (2016). Genefu: an R/Bioconductor package for computation of gene expression-based signatures in breast cancer. Bioinformatics 32 (7), 1097–1099. 10.1093/bioinformatics/btv693 26607490 PMC6410906

[B19] Goldman M. J. Craft B. Hastie M. Repecka K. McDade F. Kamath A. (2020). Visualizing and interpreting cancer genomics data via the Xena platform. Nat. Biotechnol. 38 (6), 675–678. 10.1038/s41587-020-0546-8 32444850 PMC7386072

[B20] Guo Y. Tsai H.-i. Zhang L. Zhu H. (2022). Mitochondrial DNA on tumor-associated macrophages polarization and immunity. Cancers 14 (6), 1452. 10.3390/cancers14061452 35326602 PMC8946090

[B21] Hammerl D. Massink M. P. Smid M. van Deurzen C. H. Meijers-Heijboer H. E. Waisfisz Q. (2020). Clonality, antigen recognition, and suppression of CD8+ T cells differentially affect prognosis of breast cancer subtypes. Clin. Cancer Res. 26 (2), 505–517. 10.1158/1078-0432.CCR-19-0285 31649042

[B22] Harrell Jr F. E. (2022). _rms: regression modeling strategies_. R. package version.

[B23] Home for Reasearchers (2025). Home for reasearchers. Available online at: https://www.aclbi.com/static/index.html.

[B24] Huang J. Liu M. Chen H. Zhang J. Xie X. Jiang L. (2023). Elucidating the Influence of MPT-driven necrosis-linked LncRNAs on immunotherapy outcomes, sensitivity to chemotherapy, and mechanisms of cell death in clear cell renal carcinoma. Front. Oncol. 13, 1276715. 10.3389/fonc.2023.1276715 38162499 PMC10757362

[B25] Itcho K. Oki K. Gomez-Sanchez C. E. Gomez-Sanchez E. P. Ohno H. Kobuke K. (2020). Endoplasmic Reticulum Chaperone Calmegin is upregulated in aldosterone-producing adenoma and associates with aldosterone production. Hypertension 75 (2), 492–499. 10.1161/HYPERTENSIONAHA.119.14062 31865789 PMC7004827

[B26] Kasangian A. A. Gherardi G. Biagioli E. Torri V. Moretti A. Bernardin E. (2017). The prognostic role of tumor size in early breast cancer in the era of molecular biology. PloS one 12 (12), e0189127. 10.1371/journal.pone.0189127 29211792 PMC5718505

[B27] Kassambara A. (2023). _ggpubr: 'ggplot2' based publication ready plots_. R package version 0.6.0, Available online at: https://CRAN.R-project.org/package=ggpubr.

[B8] Kerr K. F. Brown M. D. Zhu K. Janes H. Assessing the clinical impact of risk prediction models with decision curves: Guidance for correct interpretation and appropriate use. J Clin Oncol. 2016; 34 (21): 2534–40. 10.1200/JCO.2015.65.5654 PMC4962736 27247223

[B28] Kuhn M. (2008). Building predictive models in R using the caret package. J. Stat. Soft. 28(5):1–26. 10.18637/jss.v028.i05

[B29] Li J. H. Liu S. Zhou H. Qu L. H. Yang J. H. (2014). starBase v2.0: decoding miRNA-ceRNA, miRNA-ncRNA and protein–RNA interaction networks from large-scale CLIP-Seq data. Nucleic Acids Res. 42 (D1), D92–D97. 10.1093/nar/gkt1248 24297251 PMC3964941

[B30] Liu C.-Y. Zhang Y.-H. Li R.-B. Zhou L.-Y. An T. Zhang R.-C. (2018). LncRNA CAIF inhibits autophagy and attenuates myocardial infarction by blocking p53-mediated myocardin transcription. Nat. Commun. 9 (1), 29. 10.1038/s41467-017-02280-y 29295976 PMC5750208

[B31] Liu J. Zhang M. Sun Q. Qin X. Gao T. Xu Y. (2023). Construction of a novel MPT-driven necrosis-related lncRNAs signature for prognosis prediction in laryngeal squamous cell carcinoma. Environ. Sci. Pollut. Res. 30 (31), 77210–77225. 10.1007/s11356-023-26996-1 37249774

[B32] Mayama A. Takagi K. Suzuki H. Sato A. Onodera Y. Miki Y. (2018). OLFM4, LY6D and S100A7 as potent markers for distant metastasis in estrogen receptor‐positive breast carcinoma. Cancer Sci. 109 (10), 3350–3359. 10.1111/cas.13770 30137688 PMC6172070

[B33] Mohebifar H. Sabbaghian A. Farazmandfar T. Golalipour M. (2023). Construction and analysis of pseudogene-related ceRNA network in breast cancer. Sci. Rep. 13 (1), 21874. 10.1038/s41598-023-49110-4 38072995 PMC10711010

[B34] Mozdarani H. Ezzatizadeh V. Rahbar Parvaneh R. (2020). The emerging role of the long non-coding RNA HOTAIR in breast cancer development and treatment. J. Transl. Med. 18 (1), 152. 10.1186/s12967-020-02320-0 32245498 PMC7119166

[B35] NavaneethaKrishnan S. Rosales J. L. Lee K.-Y. (2018). Loss of Cdk5 in breast cancer cells promotes ROS-mediated cell death through dysregulation of the mitochondrial permeability transition pore. Oncogene 37 (13), 1788–1804. 10.1038/s41388-017-0103-1 29348461 PMC5874258

[B36] Newman A. M. Liu C. L. Green M. R. Gentles A. J. Feng W. Xu Y. (2015). Robust enumeration of cell subsets from tissue expression profiles. Nat. Methods 12 (5), 453–457. 10.1038/nmeth.3337 25822800 PMC4739640

[B37] Niu X. Zhang J. Zhang J. Bai L. Hu S. Zhang Z. (2024). Lipid nanoparticle-mediated oip5-as1 delivery preserves mitochondrial function in myocardial ischemia/reperfusion injury by inhibiting the p53 pathway. ACS Appl. Mater. and Interfaces 16 (45), 61565–61582. 10.1021/acsami.4c10032 39485791

[B38] Parker J. S. Mullins M. Cheang M. C. Leung S. Voduc D. Vickery T. (2009). Supervised risk predictor of breast cancer based on intrinsic subtypes. J. Clin. Oncol. 27 (8), 1160–1167. 10.1200/JCO.2008.18.1370 19204204 PMC2667820

[B39] Reich M. Liefeld T. Gould J. Lerner J. Tamayo P. Mesirov J. P. (2006). GenePattern 2.0. Nat. Genet. 38 (5), pp500–501. 10.1038/ng0506-500 16642009

[B40] Rodriguez-Ruiz M. E. Vitale I. Harrington K. J. Melero I. Galluzzi L. (2020). Immunological impact of cell death signaling driven by radiation on the tumor microenvironment. Nat. Immunol. 21 (2), 120–134. 10.1038/s41590-019-0561-4 31873291

[B41] Rose C. R. Blum R. Pichler B. Lepier A. Kafitz K. W. Konnerth A. (2003). Truncated TrkB-T1 mediates neurotrophin-evoked calcium signalling in glia cells. Nature 426 (6962), 74–78. 10.1038/nature01983 14603320

[B42] Rossi M. N. Antonangeli F. (2014). LncRNAs: new players in apoptosis control. Int. J. cell Biol. 2014, 473857. 10.1155/2014/473857 24627686 PMC3929073

[B43] Shannon P. Markiel A. Ozier O. Baliga N. S. Wang J. T. Ramage D. (2003). Cytoscape: a software environment for integrated models of biomolecular interaction networks. Genome Res. 13 (11), 2498–2504. 10.1101/gr.1239303 14597658 PMC403769

[B44] Shen W. Song Z. Zhong X. Huang M. Shen D. Gao P. (2022). Sangerbox: a comprehensive, interaction‐friendly clinical bioinformatics analysis platform. Imeta 1 (3), e36. 10.1002/imt2.36 38868713 PMC10989974

[B45] Slamon D. J. Godolphin W. Jones L. A. Holt J. A. Wong S. G. Keith D. E. (1989). Studies of the HER-2/neu proto-oncogene in human breast and ovarian cancer. Science. 244 (4905), 707–712. 10.1126/science.2470152 2470152

[B46] Sparano J. A. Gray R. J. Makower D. F. Pritchard K. I. Albain K. S. Hayes D. F. (2018). Adjuvant chemotherapy guided by a 21-gene expression assay in breast cancer. N. Engl. J. Med. 379 (2), 111–121. 10.1056/NEJMoa1804710 29860917 PMC6172658

[B47] Sung H. Ferlay J. Siegel R. L. Laversanne M. Soerjomataram I. Jemal A. (2021). Global cancer statistics 2020: GLOBOCAN estimates of incidence and mortality worldwide for 36 cancers in 185 countries. CA a cancer J. Clin. 71 (3), 209–249. 10.3322/caac.21660 33538338

[B48] Wallden B. Storhoff J. Nielsen T. Dowidar N. Schaper C. Ferree S. (2015). Development and verification of the PAM50-based Prosigna breast cancer gene signature assay. BMC Med. genomics 8, 54–14. 10.1186/s12920-015-0129-6 26297356 PMC4546262

[B49] Wang C. Sun D. Huang X. Wan C. Li Z. Han Y. (2020). Integrative analyses of single-cell transcriptome and regulome using MAESTRO. Genome Biol. 21 (1), 198. 10.1186/s13059-020-02116-x 32767996 PMC7412809

[B50] Wang K. Liu F. Liu C. An T. Zhang J. Zhou L. (2016). The long noncoding RNA NRF regulates programmed necrosis and myocardial injury during ischemia and reperfusion by targeting miR-873. Cell Death and Differ. 23 (8), 1394–1405. 10.1038/cdd.2016.28 PMC4947670 27258785

[B51] Wickham H. (2007). Reshaping data with the reshape package. J. Stat. Soft. 21 (12), 1–20. 10.18637/jss.v021.i12

[B52] Wickham H. (2016). ggplot2: elegant graphics for data analysis. Springer-Verlag New York.

[B53] Wilkerson M. D. Hayes D. N. (2010). ConsensusClusterPlus: a class discovery tool with confidence assessments and item tracking. Bioinformatics 26 (12), 1572–1573. 10.1093/bioinformatics/btq170 20427518 PMC2881355

[B54] Xia Q. Yan Q. Wang Z. Huang Q. Zheng X. Shen J. (2023). Disulfidptosis-associated lncRNAs predict breast cancer subtypes. Sci. Rep. 13 (1), 16268. 10.1038/s41598-023-43414-1 37758759 PMC10533517

[B55] Yang M. Lu H. Liu J. Wu S. Kim P. Zhou X. (2022). lncRNAfunc: a knowledgebase of lncRNA function in human cancer. Nucleic acids Res. 50 (D1), D1295–D1306. 10.1093/nar/gkab1035 34791419 PMC8728133

[B56] Yang S. Wang X. Zhou X. Hou L. Wu J. Zhang W. (2023). ncRNA-mediated ceRNA regulatory network: transcriptomic insights into breast cancer progression and treatment strategies. Biomed. and Pharmacother. 162, 114698–8. 10.1016/j.biopha.2023.114698 37060661

[B57] Yu J. Zhong B. Xiao Q. Du L. Hou Y. Sun H.-S. (2020). Induction of programmed necrosis: a novel anti-cancer strategy for natural compounds. Pharmacol. and Ther. 214, 107593. 10.1016/j.pharmthera.2020.107593 32492512

[B58] Zhou K. R. Liu S. Li B. Liu S. R. Zheng W. J. Cai L. (2025). An encyclopedia of RNA interactomes in ENCORI.

[B59] Zhu L. Cui K. Weng L. Yu P. Du Y. Zhang T. (2021). A panel of 8-lncRNA predicts prognosis of breast cancer patients and migration of breast cancer cells. PloS one 16 (6), e0249174. 10.1371/journal.pone.0249174 34086679 PMC8177463

